# Impact of selenium and selenoproteins on idiopathic male infertility: a comprehensive review

**DOI:** 10.3389/fnut.2025.1702028

**Published:** 2026-01-08

**Authors:** Nebojša Zečević, Ivana Šarac, Milan Perović, Milica Zeković, Aleksandar Stojsavljević

**Affiliations:** 1 Clinic for Gynecology and Obstetrics "Narodni Front", Belgrade, Serbia; 2 Faculty of Medicine, University of Belgrade, Belgrade, Serbia; 3 Special Hospital Belgrade, Human Reproduction Center, Belgrade, Serbia; 4 Centre of Research Excellence in Nutrition and Metabolism, Group for Nutrition and Metabolism, Institute for Medical Research, National Institute of the Republic of Serbia, University of Belgrade, Belgrade, Serbia; 5 Innovative Centre of the Faculty of Chemistry, University of Belgrade, Belgrade, Serbia

**Keywords:** infertility, male fertility, selenium, selenoproteins, seminal plasma, sperm quality, spermatozoa, supplementation

## Abstract

This comprehensive review systematizes the recent literature on the role of selenium (Se) and selenoproteins in male fertility and the mechanisms involved, by integrating data from animal, human, and *in vitro* studies. Additionally, it gives an overview of human studies published on the Se levels in seminal plasma worldwide, comparison of Se levels in seminal plasma and blood (serum or plasma) between infertile and fertile men, associations between seminal and/or blood Se levels and sperm quality, and elucidates whether Se supplementation could be a sustainable treatment for male infertility. Overall, the findings from studies in experimental and domestic animals, cell cultures, and humans confirm the role of Se and selenoproteins in male fertility, suggesting the multiple mechanisms involved at various levels of the male reproductive system. The studies in humans on Se seminal levels show that, generally, infertile men tend to have lower levels of seminal plasma Se; however, in some cases, significantly higher Se levels have been observed, suggesting that excessive Se may also be linked to infertility. Additionally, most studies demonstrate a positive correlation between Se levels in both seminal plasma and blood with certain seminogram quality parameters, particularly sperm motility. These findings, along with most available clinical trials, support the potential benefits of Se supplementation for improving male infertility. However, there are limited studies on Se status or supplementation concerning *in vitro* fertilization outcomes and pregnancy, as well as the association of other functional Se-status biomarkers in seminal plasma with infertility. Further studies are needed to define the best Se-status biomarkers related to male fertility, their optimal ranges, analytical techniques for their assessment, and the optimal Se supplementation dosages, formulations, and treatment durations. Additionally, the potential health risks associated with high-dose Se intakes should be carefully considered.

## 1 Introduction

### 1.1 A brief overview of the male reproductive system and male infertility

According to the World Health Organization (WHO), infertility is defined as the failure to conceive after 12 months or more of regular, unprotected intercourse. Infertility is a disease of the male or female reproductive system and is of increasing incidence in almost all countries of the world, regardless of ethnicity (1). The latest data show that approximately 15% of reproductive couples in the world have a problem with infertility (2). The causes of infertility can be related to men, women, or both partners (1, 3). Although female infertility is an important part of any infertility discussion, this manuscript focuses only on the impact of Se on male infertility. Men are solely responsible in approximately 20% of cases and a contributing factor in another 30–40% of all infertility cases (4). Worldwide data show a decline in sperm concentration (−0.64 million/mL per year) from 1973 to 2011 (5). Although the field of reproductive medicine is developing rapidly, the causes of male infertility have not been fully resolved (6). Age, contaminated environment, occupational exposure, diet, and lifestyle are predisposing factors for inadequate sperm quality (7). Specific medical diseases/conditions are also associated with male infertility, including COVID-19 (8, 9). Yet, in 30% of infertile men, the cause of infertility cannot be determined—idiopathic (unknown) infertility (10).

**Figure 1:**
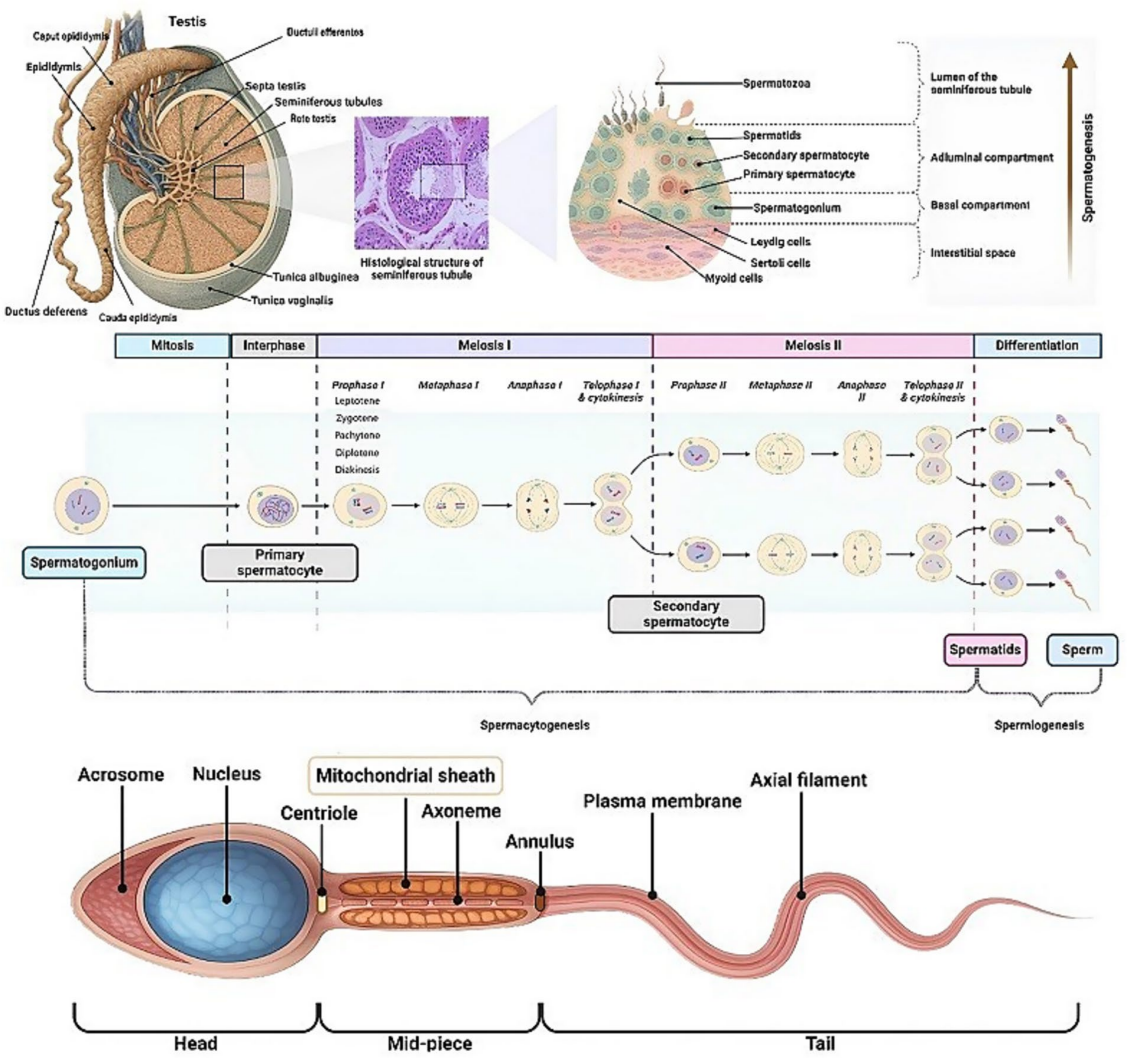
Integrated representation of human spermatogenesis: from testicular architecture, through germ cell progression, to the structure of mature spermatozoa. Note: This illustration delineates the anatomical context, histological appearance, and cellular dynamics of spermatogenesis within the seminiferous epithelium. The process initiates with spermatogonia in the basal compartment undergoing mitotic divisions, followed by entry into meiotic phases as primary and subsequently secondary spermatocytes. Replication of DNA occurs during interphase (premeiotic-S phase of the cell cycle). In prophase I of meiosis I, which is the longest step, several sub-stages are described: leptotene, zygotene, pachytene, diplotene, and diakinesis. During pachytene, the lengthiest stage, the crossing over is completed. Haploid spermatids arise post-meiosis and undergo spermiogenesis, a morphological transformation culminating in mature spermatozoa. The spatial interplay between somatic support cells (Sertoli, Leydig, and myoid cells) and germ cell populations underscores the compartmentalized architecture of the seminiferous epithelium and the orchestrated complexity of male germ cell development (236–238).

The male reproductive system encompasses the hypothalamic–pituitary–gonadal (HPG) axis, which includes parts of the brain, specifically the hypothalamus and pituitary gland, as well as the testes (11, 12). It also comprises accessory organs such as the epididymis, vas deferens, scrotum, prostate gland, seminal vesicles, seminal vesicle ducts, ejaculatory ducts, bulbourethral (Cowper) glands, urethra, and penis. These components work together in the processes of semen production, maturation, nourishment, transport, and sexual intercourse (copulation) (13, 14). The HPG axis is responsible for the production of testosterone, the main sex hormone in males, other sex steroid hormones (dehydroepiandrosterone—DHEA, androstenedione, dihydrotestosterone, estrogen, and progesterone), peptide hormones (activin, inhibin, and follistatin, which are included in feedback mechanisms and spermatogenesis), production of spermatozoa (sperm cells), and sexual arousal (11, 15). The HPG axis functions through the pulse production of the gonadotropin-releasing hormone (GnRH) from the hypothalamus, which in the pituitary stimulates the production of the follicle-stimulating hormone (FSH), necessary for sperm production (spermatogenesis), and luteinizing hormone (LH), necessary for both testosterone production and spermatogenesis (11, 15). Within the testis, the main cells involved in the production of testosterone and spermatogenesis are the interstitium-located Leydig cells and the seminiferous tubules-located Sertoli cells and germ cell lines. Leydig cells produce mainly testosterone and less insulin-like growth factor 3 (IGF-3). In contrast, Sertoli cells produce mainly estradiol, inhibin B, activin A, anti-Müllerian hormone (AMH), and IGF-3, which control steroidogenesis by a feedback loop. Additionally, Sertoli cells nourish, support, and protect spermatogonia and the developing sperm cells (15). During spermatogenesis, from spermatogonia, the mature haploid sperm cells are formed, through subsequent stages of mitosis, meiosis, differentiation, and maturation (“spermiation”) in the testes’ seminiferous tubules. This process includes the formation of intermediate primary and secondary spermatocytes and spermatids, until the mature sperm cells are formed and released from Sertoli cells into the lumen of seminiferous tubules, the rete testis, and efferent ducts (16, 17). Interstitial contractile myoid cells, by their contraction, are involved in the transport of spermatozoa and testicular fluid in the tubules. The epididymis stores, protects, and concentrates the spermatozoa before ejaculation, and provides an environment for further maturation (in the caput epididymis), during which spermatozoa become motile (in the cauda epididymis) and with fertilizing capacity (18). A schematic illustration of male reproductive organs, the structure of seminiferous tubules, and the sequential stages of spermatogenesis is given in Figure 1.

Additionally, other endocrine parts of the body (including the adrenal glands, thyroid gland, pancreas, and fat tissue) communicate with the HPG axis and accessory organs to achieve adequate production of sex hormones and sperm quality and quantity. For example, in fat tissue, the interconversion of various sex hormones takes place, while fat tissue also secretes various adipokines, cytokines, and fatty acids that can directly or indirectly modulate the production of sex hormones and spermatogenesis (19–21). Insulin and thyroid hormones are also potent modulators of the HPG axis (22–25). Adrenal glands secrete the adrenal sex hormones (androstenedione, DHEAS), which in the adipose tissue could convert to more potent testosterone and exert a negative or positive feedback to the HPG axis. In post-pubertal men, more than 95% of total circulating testosterone derives from the testes, while the remainder derives from the adrenals (12).

Human semen is a complex secretion and consists of two main components: testicular secretion (containing sperm/sperm cells/spermatozoa, which make up approximately 5% of the total volume) and seminal plasma/fluid (the remaining 95%) (26). Seminal plasma is a rich mixture of secretions from the accessory sex glands (seminal vesicles, prostate, and bulbourethral gland), the lumen of the seminiferous tubules, epididymis, and vas deferens (27). The seminal vesicles contribute approximately 65–75% of the seminal plasma, while the prostate contributes approximately 20–30%. The remaining 10% originates from the bulbourethral glands, the lumen of the seminiferous tubules, the epididymis, and the vas deferens (28). Seminal plasma regulates sperm capacitation (the process by which sperm become capable of fertilizing a mature egg) and their interaction with female secretions within the female reproductive tract. Moreover, seminal plasma helps protect and mature sperm, since mature spermatozoa are transcriptionally and translationally inactive, with a condensed nucleus (DNA/chromatin tightly packed and protected with protamines and disulfide bonds) and with reduced cytoplasm and no ribosome content (29) (Figure 1).

Basic semen analysis (seminogram/spermiogram) is still considered fundamental in the evaluation of male infertility. However, despite being routinely used, semen analysis cannot fully distinguish infertile from fertile men (30). As a consequence of intra-individual variability, the WHO recommends performing two or three seminograms to obtain in-depth information about the patient’s seminal/semen parameters (WHO, 2024). However, finding new biomarkers that could distinguish fertile from infertile men is of crucial importance (31).

Normal seminogram findings encompass ejaculate volume > 1.5 mL, sperm count > 40 mil./ejaculate, sperm concentration > 15 mil./mL, a viability of > 54% live sperm, total motility of 40%, with 30% having progressive motility, and > 4% having normal morphology. The WHO suggests employing the following terms in clinical practice to define seminogram findings: a normal finding according to recommended WHO parameters (normozoospermia, see above); reduced sperm motility (asthenozoospermia); low sperm cell count/concentration (oligozoospermia); reduced proportion of morphologically normal sperm cells (teratozoospermia); absence of sperm cells in the ejaculate (azoospermia) (32, 33). Approximately 45% of men with infertility are affected by either oligozoospermia or azoospermia. These conditions are commonly related, such as in oligoasthenozoospermia (34).

Male infertility can result from inadequate spermatozoa concentration or absence, and aberrant spermatozoa morphology and motility due to testicular failure (Klinefelter’s syndrome, chemotherapy, radiation therapy, undescended testicles, or varicocele—enlargement of the venous plexus in the scrotum), hormonal imbalances and use of anabolic steroids, detection of antisperm antibodies, sexually transmitted diseases, or ejaculatory obstructions, including cystic fibrosis (often presenting with bilateral absence of the vas deferens) (35, 36). Additional risk factors encompass age, obesity (BMI ≥ 30), inadequate diet, alcohol consumption, anxiety and depression, working in extremely hot conditions, the presence of Cushing’s syndrome, mumps, hypospadias, anemia, diabetes, metabolic syndrome, and thyroid diseases (37, 38).

### 1.2 A brief overview of the role of Se and selenoproteins in the human body, symptoms of Se deficiency and excess, dietary Se sources and forms, recommended daily Se intakes, worldwide intakes, and biomarkers of Se status

Selenium is an essential trace element for human health. Its effects are largely mediated by selenium-containing proteins (selenoproteins), which contain at least one selenocysteine (SeCys/Sec), a selenium-containing amino acid instead of sulfur (S) (39, 40). The human genome contains 25 genes for different selenoproteins, all of them containing only one SeCys, except selenoprotein P (SELENOP/SelP/SEPP1), which contains 10 SeCys. Most of them exert redox potential control and oxidoreductase function, e.g., glutathione peroxidase (GPX/GPx) 1–4 and 6, thioredoxin reductase (TXNRD/TrxR) 1–3, and selenoprotein R/methionine sulfoxide reductase B1 (SELENOR/SelR/MSRB1). Others are included in the synthesis and activation of thyroid hormones, e.g., iodothyronine deiodinase (DIO) 1–3, synthesis of selenoproteins, e.g., selenophosphate synthetase 2 (SPS2/SEPHS2), or transport of Se (e.g., selenoprotein P) (39, 40). The role of different selenoproteins in the male reproductive function will be discussed in more detail later, in Sections 2 and 3.

Apart from female and male infertility (41, 42), Se deficiency was connected to heart disease (possibly fatal congestive dilated cardiomyopathy, including endemic Keshan disease), musculoskeletal and neuromuscular disorders (e.g., Kashin–Beck disease), thyroid function dysfunction, disrupted immune function, poor response to acute and chronic infections (including COVID-19, Coxsackie, influenza, and HIV viral infections), chronic inflammation, cancer (including colonic, gastric, pulmonary, and prostate carcinoma), and other disorders (40, 43–48). In contrast, excessive Se intake or environmental exposure was associated with an increased risk for type 2 diabetes, neurodegenerative diseases (amyotrophic lateral sclerosis and Parkinson’s disease), and increased risk and mortality from some malignancies (carcinoma of prostate, buccal cavity, pharynx, stomach, colon, rectum, lungs, kidney, and urinary bladder, melanoma, multiple myeloma, lymphoid neoplasms, etc.) (43, 49, 50).

Dietary Se is obtained from a wide range of food sources, including meat (particularly offal—kidney, liver, testicles, hearts, lungs, and brain), fish, crustacean, eggs, milk/dairy products, nuts, vegetables, legumes, bread, and cereals, as well as from drinking water, but in a much lesser amount (51). Although the richest dietary Se sources are Brazil nuts, offal (kidney), fish, and crustacean, the main dietary Se contributors in the European diet are milk and dairy products, meat and meat products, grains and grain-based products, and less fish and fish products (51). Additionally, Se-enriched dietary products and Se supplements can be important Se sources. In the diet and water, it can be present in the organic form (including SeCys and SeMet, where Se replaces S in the methionine (Met) and cysteine (Cys), respectively, and methylselenocysteine—MeSeCys) and inorganic form (selenate, selenite, selenide, and elemental Se) (45, 51–53). Selenate and selenite are the two major forms of Se in soil, with selenate predominating in oxic soils and selenite predominating in anoxic soils (54), and plants absorb these inorganic forms and convert them to organic forms. Selenite is more quickly converted than selenate, through the mid-phase of selenide before making SeCys, while selenate needs to be first converted to selenite (54). Organic selenocompounds can also be present in some soils and absorbed by plants (54). In general, the main form of Se that humans consume through ingestion is SeMet (55, 56), which is also the predominant Se compound in animals and humans. The second most abundant dietary source is SeCys, while selenate and selenite represent the major inorganic sources, ingested mostly through water or supplements, less through plants and animals (e.g., fish), and contribute less to the daily dietary intakes (45, 57). Most forms of Se are effectively absorbed in the gastrointestinal tract (nearly 100% for selenate, > 90% for SeMet, and 80% for selenite) (55). However, the absorption of Se also depends on the content of protein, fat, heavy metals, and other compounds in the diet, and food preparation practices (55), and from the average diet, the Se absorption is approximately 70–80% (45, 57). The retention of the absorbed organic Se forms is higher than that of inorganic forms, which are easily lost through urine (particularly selenate) (55). According to some reports, the bioavailability of Se from SeMet in poultry is almost twice that from selenite (71% vs. 36%), and its biological potency is much higher (45, 52). Absorption and retention of Se from Se-enriched yeast supplements vary from 50 to 95% (56). Bioactivity of Se-enriched animal food (milk and meat) is higher than that of pure SeMet (58). In Brazil nuts, the major Se form is SeMet, and the bioavailability of Se from Brazil nuts is also higher than the bioavailability of pure SeMet (55). Interestingly, Se content in Brazil nuts ranges widely, from 0.03 μg/g to 512 μg/g (wet weight) depending on location; nuts from trees in central Brazil have ≤ 10-fold more Se than those from western Brazil (55, 56, 59). Since the dietary Se content in Brazil nuts varies and could overcome the tolerable upper intake limits, it would be indicative to introduce legislative measures for mandatory nutritional labels displaying their Se content and the allowed daily consumption amount. Other Se-rich food sources, so-called “Se accumulators,” are Brassica species (rapeseed, broccoli, and cabbage) and Allium species (garlic, onion, leek, and wild leek), which contain mostly MeSeCys and less SeMet and selenate. In contrast, cereals and legumes (soya) contain mostly SeMet, and less SeCys and selenate (54, 60). Other plant foods mostly contain SeMet (approximately 90% of Se content). There are also “Se hyper-accumulators”—a class of non-edible plants, such as Astragalus and *Stanleya pinnata* species, which are not edible, able to accumulate and tolerate concentrations of Se approximately 1,000 times higher than normal plants, due to high conversion rate to MeSeCys, *γ*-glutamyl-MeSeCys, dimethylselenide (DMSe), and dimethyldiselenide (DMDSe) (54). The Se content of foods from animal sources varies according to the diet of the animals and can be presented mostly as SeCys and SeMet, much less selenate, selenite, selenide, and other organic Se compounds (e.g., selenoneine in fish and marine mammals). Dairy products contain mainly SeCys, SeMet, and other Se organic compounds, and SeCys in the milk is mainly in the form of GPX, Se-cystamine, Se-cystine (SeCys2) (61–63). In the water, selenate is the major form but is usually in negligible concentrations (45).

The recommended daily intake of selenium (Se) varies based on different guidelines. The World Health Organization (WHO) and the Food and Agriculture Organization of the United Nations (FAO) suggest an intake of 34 μg for adult men and 26 μg for adult women. In contrast, the US Institute of Medicine (IOM) and the European Scientific Committee on Food (SCF) recommend an intake of 55 μg for both genders. Finally, the European Food Safety Authority (EFSA) has set an even higher recommendation of 70 μg (45, 64). However, Se has a narrow range of safety intake, because of the risk of acute and chronic toxicity (“selenosis”) and its potential carcinogenicity (51). Initial signs of Se excess are a garlic-like breath odor and a metallic taste in the mouth, followed by gastrointestinal symptoms (diarrhea, nausea, and vomiting), fatigue, rash (dermatitis), hair and nail damage and loss, and teeth damage and discoloration. Se excess can further lead to joint pain, muscle weakness, tremor, muscle spasms, ataxia, headache, confusion and memory loss, and, in more severe cases of intoxication, fever, hypotension, abnormal electrocardiogram, cardiac arrest, respiratory arrest, liver toxicity, delirium, coma, and death (51, 65, 66). For those reasons, the upper tolerable limit for daily Se consumption to prevent chronic toxicity was set to 300–450 μg by various guidelines, including the IOM, SCF, WHO/FAO, and the UK Expert Group on Vitamins and Minerals (51). For example, the tolerable upper intake of Se is set at 400 μg/day by the guidelines of WHO/FAO and some countries (e.g., the US, Australia, New Zealand, and China) (67). However, according to the EFSA, the risk of developing alopecia, an early sign of chronic Se toxicity, is increased even with Se intakes of 330 μg/day (130 μg/day from the background diet plus 200 μg/day from supplements) (51). Furthermore, in light of more recent data on the possible effect of Se on insulin resistance and the risk for type 2 diabetes, the upper tolerable limit has been further reduced to 255 μg/day by the latest EFSA recommendations from 2023 (51). Nevertheless, this dietary intake is hard to exceed, except for consumers who, in their diet, regularly consume Brazil nuts or high-dose Se supplements, not complying with the recommendations (51). More specifically, some authors (Vincenti and colleagues) recommend separate limits: 260 μg/day for organic Se (selenomethionine, SeMet) and 16 μg/day for inorganic hexavalent Se (selenate), as the experimental evidence *in vitro* and in animals suggests a much higher toxicity with selenate compared with organic Se. Since selenate is typically found in waters, these authors also propose Se limits of 1 μg/L in drinking water (while the EU and WHO regulations allow 20–40 μg/L) (49, 68).

Dietary Se intake clearly differs between regions of the world, largely due to the variable Se content of soil, as well as pH, redox, and water content of the soil, which all influence the Se uptake by growing plants, and consequently, animals exposed, as well as water and the type of food consumed by humans (45, 69). For example, in the USA, a population study reported an average daily Se intake of 106 ± 50.7 μg, corresponding to serum Se levels of 137 ± 18.9 μg/L (70). On the other hand, daily Se intake in Europe is notably lower than in the USA (11–70 μg per day) (56, 64). Eastern Europe and Northern Europe (e.g., Nordic and Baltic countries, Poland, Belarus, and Ukraine) have the lowest Se intake: 7–30 μg/day and 22–88 μg/day, respectively (note: in Finland, since 1984 there has been an addition of Se to fertilizers; in Sweden, since the late 1980s, there is addition of Se to animal feed; in Norway and Iceland, there is an import of high-Se wheat from North America). On the other hand, Central, Southern, and Western Europe had various daily Se intakes (30–90 μg/day), being the highest reported in France, Belgium, Spain, and lower in the UK, the Balkans, Austria, Hungary, Germany, and Italy (56, 57, 64). In some parts of China (e.g., Keshan), the intake was only 3–14 μg/day (56, 57), and the endemic Se deficiency appeared in the form of chronic osteochondropathy (Kashin–Beck disease) and chronic cardiomyopathy (Keshan disease), which are now preventable through Se supplementation (71, 72). In contrast, in some other Chinese provinces (e.g., Enshi), Se intake was very high (2.144–6.69 mg/day), and endemic selenosis outbreaks appeared (56, 73). Other Se-deficient areas are some parts of Russia, New Zealand, Australia, the Middle East, South Asia, Ethiopia, and sub-Saharan Africa (45, 57, 74).

In the Se-deficient areas, at the highest risk are people who consume locally organically grown plants and animal products from animals fed local organically grown feeds. Additionally, vegetarians, particularly vegans, are at a higher risk for Se deficiency, because plant foods might contain low levels of Se, while in some countries, animal feed is fortified with Se (56, 57, 69).

The WHO recommends serum Se levels in the range from 39.5 to 197 μg/L. However, considering that maximal GPX activity occurs at 70–90 μg/L, and that maximal selenoprotein P levels occur closer to 120 μg/L, many recent studies have suggested a narrower Se range of 70–130 μg/L (45, 57) (Note: 100 μg/L equals 1.266464 μmol/L, to convert to Standard International Units).

Serum/plasma total Se concentrations can be influenced by sex, age, smoking, water and diet Se content, dietary sources of Se (organic or inorganic), and various conditions that increase its demands or decrease its intake/utilization, leading to decreased Se concentrations (e.g., inflammation, anorexia, intestinal malabsorption syndromes, and total enteral or parenteral nutrition without adequate Se dosing) (45, 57, 75). In countries with high Se concentrations and availability in the soil (e.g., USA), the average Se levels in serum/plasma were reported to be ~122–137 μg/L, while in countries with decreased Se content in the soil, the levels were ~58–70 μg/L (e.g., in New Zealand and Finland before fertilization of the soil) (57, 64, 76). In Europe, the Se concentrations ranged from 48–124 μg/L, with mean values of 75–110 μg/L (45, 69).

Serum/plasma/whole blood Se pool is insatiable. It includes various selenoproteins with the functional SeCys, including selenoprotein P and extracellular glutathione peroxidase GPX3 in the serum (which account for ~30–60% and 10–30% of serum Se, respectively), and GPX1 in leukocytes, platelets, and red blood cells. However, there are also other proteins with non-specifically incorporated SeMet (e.g., plasma albumin) and selenosugars. Therefore, total serum/plasma/whole blood total Se levels are not proper biomarkers of the functional Se pool in the body (45).

Measured GPX activities in the plasma (GPX3) or blood cells (GPX1 and GPX4) can be used as better biomarkers of the functional Se status. However, the activities of GPXs attain a plateau with Se intakes that are lower than those required for plateauing the levels of selenoprotein P, which is considered the most informative biomarker of the functional Se status (45).

Selenoprotein P is synthesized mostly in the liver (75%), but its plasma levels reflect the functional Se status in the whole organism (57). It is (in general) fully plateaued with daily intake of 100 μg and serum/plasma levels of 70–130 or 90–140 μg/L (depending on the study), and the concentrations of ~5.5 mg/L (measured by ELISA) were associated with the Se sufficiency (e.g., among the USA population). In contrast, in the Se-deficient regions (e.g., among the Chinese Se-deficient population), the levels were only ~2.0 mg/L (45, 57). Apart from delivering the Se from the liver to other parts of the body, it also exerts enzymatic antioxidant (peroxidase) activity in plasma, notably against peroxynitrite-mediated oxidation, nitration, and nitrosation (77–79), and against lipid hydroperoxides formed by lipoxygenases and cyclooxygenases, thus protecting circulating lipoproteins (phospholipids) and cholesterol from oxidation (57, 80–82). However, it is also associated with insulin resistance and impaired insulin secretion, and thus, risk for type 2 diabetes by inhibition of AMP-activated protein kinase (AMPK) and impairments of pancreatic *β* cells function (51, 83, 84). Additionally, its higher levels were associated with metabolic syndrome, nonalcoholic fatty liver disease (NAFLD), cancer, and pulmonary arterial hypertension (PAH), but with more controversy (80, 84).

Other biomarkers of Se status, including Se urinary excretion or Se concentration in hair and (toe)nails, are less confirmed to be associated with the functional Se status (45, 80, 84). Most of the total body Se pool (which is ~5–20 mg in the European and US population) is stored in the muscle (30–50%), bones (15%), blood (10%), liver (8%), kidneys (3%), and brain (3%) (45).

## 2 Role of Se and associated selenoproteins in male fertility—an overview and main molecular mechanisms

Selenium is a critical trace element for testicular development, spermatogenesis, semen quality, testosterone biosynthesis, and, ultimately, male fertility (41, 85–87). Both low and high levels of Se in semen negatively affect sperm quality (a bell-shaped curve) (88). That Se is essential for testicular function was first confirmed by the studies in mice in which the selenoprotein P gene was knocked out (85, 89).

The testicles contain high levels of Se (90), 274 ± 48 ng/g wet weight, and are among the organs in the body with the highest Se concentrations (together with the thyroid gland, kidneys, and liver, followed by spleen, heart, lung, prostate, brain, muscle, pancreas, and gastrointestinal tract), containing double amounts compared to other parts of the male reproductive tract (epididymis, prostate, and seminal vesicles) (57, 91). In spermatozoa, 0.79 and 0.14 femtograms (i.e., 10–15 g) of Se are present in the midpiece and head of spermatozoa, respectively, with ∼5.6 times higher Se levels and 18 times lower Se concentrations in the midpiece than in the head (85, 92). However, in the human semen, less than 15% Se originates from spermatozoa, while more than 85% originates from seminal fluid (88). This is in accordance with a little difference in total semen Se of azoospermic and vasectomized men (with no spermatozoa), compared with fertile men (88). The semen Se concentrations in infertile men hugely varied in that study, between 7 to 230 ng/mL, while in proven fertile men were 67.4 ± 5.4 ng/mL. In accordance, the best seminogram regarding motility and the highest pregnancy rates during *in vitro* fertilization (IVF) were achieved with semen Se range of 60–70 ng/mL, while the worst IVF success and all seminogram characteristics were associated with the semen Se < 35 ng/mL (88). Of note, the sperm count and concentration were in positive association with the semen Se levels (88).

As part of selenoproteins, Se mediates the adequate structural integrity of sperm and exerts an antioxidant role. Some of the selenoproteins are highly expressed and more specific for testicles, e.g., certain forms of GPX4 (see below) and TXNRD3 (45). Selenium is delivered to the testicles by the selenoprotein P and the apolipoprotein E receptor 2 (apoER2)-mediated uptake (mainly in Sertoli cells, less in Leydig cells), and is incorporated into the selenoproteins included in sperm maturation (mainly GPX4 and TXNRD3) (41, 85–87, 89). Knock-out of selenoprotein P in mice reduces the quantity of Se in the testis by 80% (89, 93).

A proper amount of reactive oxidative species (ROS), including peroxides, is required for protein–protein disulfide bridging in the sperm nucleus, sperm plasma membrane, midpiece, and flagellum, which is necessary for sperm nuclear condensation and preservation, sperm motility, capacitation, and acrosome reaction (94–96). The adequate formation of disulfide bonds is necessary to maintain the morphology and function of the mature spermatozoa, protect the nucleus from oxidative damage, and ensure their motility and fertilizing ability (96). Both GPX4 and TXNRD3 can work together to promote the formation of disulfide bonds (95), which may seem surprising, since thioredoxin (TNX)/TXNRDs and the glutaredoxin (GRX)/GPXs systems are generally involved in the reduction of disulfide bonds (97, 98). However, the structural specificities of TXND3 and GPX4 allow their interaction and joint oxidative role in disulfide bond formation (see later) (99).

Various GPXs are important for the antioxidant defense of the epididymis and ejaculated sperm cells, while GPX4 has also been recognized as critical for the proper architecture of the sperm midpiece, and in the testes, three isoforms were discovered (100–102). GPX4 (also known as phospholipid hydroperoxide glutathione peroxidase, PHGPX) is not only a phospholipid-bound hydroperoxide glutathione peroxidase in the intracellular membranes and cytoplasm (cytoplasmic GPX4 isoform, cGPX4). It is also a key structural sperm protein in the mitochondria of the sperm midpiece capsule (mitochondrial GPX4 isoform, mGPX4/“mitochondrial capsular protein”—MCP), and has a role in condensation of chromatin during spermatogenesis in sperm nucleus (nuclear GPX4, nGPX4/ sperm nucleus-specific GPX4, snGPX4 isoform), due to its additional disulfide isomerase/thiol peroxidase activity, particularly when there is no enough glutathione (GSH) (18, 45, 85, 100). These three isoforms of GPX4 are produced from the same gene through alternative splicing and transcription, but have distinct histological and cellular locations and main functions (103).

While homozygous total GPX4 deletion is lethal in embryo, spermatocyte-specific GPX4 knock-out mice are infertile, with decreased number of spermatogenic cells in seminiferous tubules and spermatozoa in epididymis, reduced spermatozoa motility (absence of forward motility), reduced mitochondrial membrane potential, mitochondrial swelling, and midpiece morphological changes with tail bending in a hairpin form, similarly as in the severe Se deficiency (104–107). The tail bending in a hairpin form was also observed in spermatozoa of selenoprotein P or ApoER2 knock-out mice and mice long-term fed a Se-deficient diet, together with other abnormalities seen in the Se deficiency (85, 93, 108–110). It is an interesting finding that only mGPX4 knock-out mice are infertile with a hairpin-shaped tail, mitochondrial swelling, tail and midpiece disorganization, impaired motility, progressivity, and fertilizing ability (as described above), while nGPX4 knock-out mice have normal fertility, even though defects in chromatin condensation and sperm head abnormalities were found (100, 111, 112). While cGPX4 is a predominant isoform in somatic tissues, mGPX4 is a predominant isoform in the testes, and mGPX4 knock-out mice have a 60% lower Se level in testes (92, 103). Both mGPX4 knock-out mice and nGPX4 knock-out mice have an increased protein thiol content in spermatozoa from cauda epididymis and vas deferens (103, 104, 111, 113). In men, genetic polymorphisms of *gpx4* were weakly associated with seminal plasma GPX activity (possibly because GPX4 is not a major GPX in seminal plasma, see later), but may account for infertility in exceptional cases (89).

The GPX4 mRNA expression and Se incorporation were not detected in spermatogonia and early spermatocytes, and they first appeared in late primary spermatocytes (during the pachytene phase of meiosis, Figure 1), gradually increasing and reaching a peak in elongating spermatids, after which they declined to become weakly detected or completely undetected in the ending spermatozoa (114, 115). GPX4 changes from being an active peroxidase in spermatogenic cells to an inactive structural protein in spermatozoa (102). In spermatocytes and spermatids, it has active peroxidase activity that helps protect cell membranes from free radicals. In testis spermatozoa, only ~50% of the capsule protein is composed of active GPX4 (89), which neutralizes ROS generated during the mitochondrial electron transport chain redox reactions. In caudal epididymal spermatozoa, it becomes a completely inactive structural protein of the mitochondrial capsule, cross-linked to high molecular mass complexes with other capsular proteins (85, 89, 100, 102, 103). However, its activity could be restored and become even higher than in immature spermatozoa under certain conditions, including capacitation and fertilization (102, 103). Germ cells and more immature spermatogenic cells (namely type B spermatogonia and primary spermatocytes) contain the most soluble cGPX4 (116). In contrast, the nGPX4 insoluble form with thiol-oxidase activity is present in late spermatids and spermatozoa, and it affects sperm maturation and chromatin condensation through the formation of disulfide bridges in chromatin (41, 85). GPX4 expression is strongly dependent on the gonadotropin stimulation and is expressed only after puberty, disappears after hypophysectomy, and is partially restored by gonadotropin treatment (116). Testes have 20 times higher GPX4 activity compared with other tissues (e.g., liver) (116), and in Se deficiency, the activities of selenoproteins in the testis are less affected, compared with other tissues (e.g., liver), showing a hierarchy in Se delivery by selenoprotein P (93, 117).

Since mature spermatozoa contain only a small amount of endogenous antioxidants (both enzymes and non-enzymatic compounds), they rely on antioxidants in epididymal and seminal fluid secreted by the epididymis and other accessory glands (18, 95, 118). Other GPXs (1, 3, 5, and 6), together with cGPX4 (which is also present in the epididymal epithelium), are involved in the epididymal anti-oxidative defense to protect the epididymal parenchyma and maturing sperm from oxidative stress and to promote further sperm maturation, gain of motility, and other functions (18, 85, 95, 119, 120). GPX5 is Se-independent enzyme, but works in conjunction with GPX3 to protect against oxidative stress, and is exclusively expressed in epididymal caput epithelium (18, 85, 95, 121). GPX1 and GPX3 are located in the epididymal, prostate, and seminal vesicles epithelium (18, 85, 119), while GPX1 is also found in the Leydig cells (122, 123). GPX3 expression in the epididymis increases from the caput to the cauda, in contrast to GPX5, which is only found in the caput (124). GPX3 and GPX5 make up 95% of GPX in epididymal epithelium and lumen, while the rest represents cGPX4 and GPX1 in epithelium cytoplasm, and GPX6 in epithelium cytoplasm, lumen of epididymis, and spermatozoa (18, 120). In the epididymis, total GPX activity is two times higher in the cauda than in the caput, and is mostly related to GPX3 (95). With ageing, GPX5 knock-out male animals are prone to have reduced reproductive potential (represented by higher embryonic and early postnatal lethality and embryonic defects, associated with higher DNA defects in spermatozoa), while GPX1 and GPX3 knock-out male animals are completely fertile (18, 85, 95, 119, 125, 126). Similar to GPx5, GPX6 is a close homolog to GPX3, resulting from a tandem duplication of the GPx3 gene, but its role in male fertility is less examined, and it is probably involved in the prevention of premature capacitation and acrosome reaction, possibly by reducing ROS formation (120, 127). In case of a deficit of one GPX, there is a compensatory upregulation of other GPXs and other antioxidative and disulfide bond-forming systems (e.g., catalase, glutathione S transferases, peroxiredoxins, thioredoxins, and disulfide isomerases) (95, 112).

Similar to nGPX4 and mGPX4, TXNRD3 has the highest expression in the testis and is particularly abundant at the site of mitochondrial capsule formation in elongated spermatids, but is absent in mature spermatozoa (99, 100, 128, 129). Due to its thiol-oxidase activity, it is involved in the isomerization of proteins and inter-protein disulfide bonds formation, including chromatin condensation (together with nGPX4) and mitochondrial capsule formation (together with mGPX4). Interestingly, TXNRD3, unlike the other two TXNRD isozymes (1 and 2), contains an additional N-terminal GRX domain, which allows it to participate in both TXN and GRX/GSH systems. This probably explains why only this TXNRD3 isozyme can be involved also in the formation of disulfide bonds, not only in their reduction, by its interaction with GPX4 (99). TXNRD3 knock-out animals are viable, but with reduced fertility, reduced sperm count, motility, and IVF success, and they show functional and structural changes in spermatozoa during sperm maturation and capacitation, with altered thiol redox status in the head and tail and defective mitochondrial ultrastructure and activity under capacitating conditions (128, 129).

Selenoprotein V is discovered in the testes of rodents, but its role is still unknown, possibly being involved in redox regulation and regulation of other selenoprotein expression in the testis (GPX1, TXNRD3, selenoprotein P, etc.) (130).

Selenoprotein P has also antioxidative properties and is found in seminal plasma, although in much lower concentrations compared to blood plasma (approximately ten times). Seminal plasma selenoprotein P concentrations correlated positively with sperm density and vital sperm fraction (131).

Interestingly, selenoprotein P concentrations in the seminal plasma of vasectomized men were similar to controls, indicating that accessory sex glands are a testis/epididymis-independent source of selenoprotein P (131). Similarly, another study has shown that GPX enzyme activities in seminal plasma were also not lower in vasectomized men, suggesting that they did not originate substantially from the testis or epididymis, but more from the prostate, seminal vesicles, Cowper glands, and their excretory ducts (132). This is in accordance with the finding that the measured tissue GPX activity in the epididymis was lower than in the seminal vesicles or prostate. Nevertheless, in that study (132), it was not specified which GPX isozyme activity was detected. However, in another study, by using specific antibodies, it was demonstrated that most of the enzymatic activity detected in the seminal plasma of both healthy and infertile males derives from GPX3 (133). Interestingly, in a third study (134), GPX activity (determined by the oxidation of GSH) was approximately two-fold higher in seminal plasma than in serum, regardless of whether men were fertile or infertile.

The systematized overview of the role of different selenoproteins in the male reproductive tract and sperm maturation is given in Table 1, with their names, abbreviations, anatomical, histological, and intracellular expressions, specific functions, and the effect of their elimination (genetic knockdown) on male fertility.

**Table 1:** The overview of the role of different selenoproteins in the male reproductive tract: their nomenclature, histological and intracellular expression, function, and the effect of deficit (genetic knockdown) on male fertility.

Name	Abbreviation	Localization: organ (compartment in organ, i.e., cell lines); compartment in cell	Function	Knock-out effect on fertility
Glutathione peroxidase 4, mitochondrial (long-form)	mGPX4 (lGPX4)	Testis (spermatocytes, spermatids, spermatozoa; in Leydig cells, weakly expressed or almost absent); mitochondrial localization. ~80% of GPX4 in the testis.	Antioxidant role in spermatocytes and spermatids. Structural role in mature spermatozoa: (in mitochondrial capsule, making at least 50% of the capsule material).	Infertility, reduced number of spermatozoa, decreased motility, abnormal morphology of spermatozoa (tail bending in a hairpin form, mitochondrial swelling, sperm heads detached from the midpiece, the sliding of mitochondria along the midpiece, and extrusion of microtubules and outer dense fibers from the tail). Moderate morphological changes in the testis and epididymis. 60% reduction of Se in the testis.
Glutathione peroxidase 4, nuclear (sperm nucleus-specific)	nGPX4 (snGPX)	Predominantly in the testis (mostly in spermatids); nuclear localization. ~14% GPX4 in testis.	Role in nuclear condensation/ stabilization in spermatids (involved in protamine cross-linking by formation of bisulfide bonds).	Normal fertility. No change in concentration, motility, but abnormal head morphology of spermatozoa (delayed chromatin condensation, reduced disulfide bonds, incompletely compacted nuclei). No change in Se concentration in the testis. No morphological changes in the testis and epididymis.
Glutathione peroxidase 4, cytoplasmic (short-form)	cGPX4 (sGPX4)	Predominantly in somatic tissues, including epididymis, much less in testis (mainly in less mature spermatogenic cells, namely, type B spermatogonia and primary spermatocytes, not in Sertoli or Leydig cells); localization in intracellular membranes and cytoplasm, inner and outer mitochondrial membrane. ~6% GPX4 in testis.	Predominant cGPX in somatic cells. Anti-oxidative role, protects from apoptosis; essential for survival.	Lethal.
Glutathione peroxidase 1	GPX1	Predominantly in somatic tissues, including epididymis, prostate, epithelium, seminal vesicles, testis (Leydig cells); cytoplasmic localization.	Antioxidant role in parenchyma, epididymal fluid, and seminal plasma.	Normal fertility.
Glutathione peroxidase 3	GPX3	Extracellular; blood and seminal plasma; secreted to blood mainly from the kidney, and to seminal plasma from accessory glands (epithelial cells of epididymis, vas deferens, prostate, seminal vesicles, bulbourethral glands). Little is found in other tissues and the testis (epithelial cells of the efferent ducts); cytoplasmic localization in the epithelial cells, but is also secreted into the lumen.	Antioxidant role in epididymal/seminal fluid: protects mature spermatozoa from mitochondrial damage and lipid peroxidation. Regulated by androgens.	Normal fertility.
Glutathione peroxidase 5	GPX5	Epididymal caput (epithelium)-specifically expressed; cytoplasmic localization in the epithelial cells, secreted in the epididymal lumen.	Se-independent (SeCys-free) enzyme, secreted from the epithelium into the lumen in the epididymal caput. Protects against oxidative stress in conjunction with GPX3. Regulated by androgens.	Normal fertility at a young age, but with ageing it declines (lower embryo viability, higher incidence of miscarriages and developmental defects). Reduced DNA compaction, oxidative damage to DNA. Compensatory increased GPX3, GPX1, cGPX4, and catalase expression in epididymal epithelium. Oxidative injuries in the cauda.
Glutathione peroxidase 6	GPX6	In the testis, epididymis, seminal vesicle, prostate, and bulbourethral glands; in seminal plasma (more), and sperm (less); intracellular and extracellular localization; in the sperm acrosome (only before capacitation), tail, and neck area.	Se-independent enzyme in animals, while in humans, it is Se-dependent. Prevents premature capacitation and acrosome reaction of sperm (by its antioxidant role). Protein content in the seminal plasma and sperm negatively correlated with litter size, live litter size, and fertility.	Normal fertility (?), still under investigation.
Thioredoxin-glutathione reductase	TXNRD3 (TrxR3)	Testis (spermatids); intracellular localization, associated with mitochondria and nucleus.	Roles in nuclear condensation, antioxidant activity, mitochondrial protection, and formation of the mitochondrial capsule (disulfide bond formation) in spermatids. Works with nGPX4 to create disulfide bonds in protamines.	Reduced fertility, reduced sperm count, motility, and *in vitro* fertilization success. Reduced disulfide bond formation, defective nuclear condensation, and midpiece bending. Defective capacitation. Defective nuclear and mitochondrial ultrastructure and activity during capacitation.
Selenoprotein P	SELENOP (SelP, SEPP1)	Liver, testis (only in Leydig cells), blood (secreted from the liver), seminal plasma (secreted from the prostate, seminal vesicles, bulbourethral glands, and epididymis); cytoplasmic and extracellular localization.	Role in Se-transport from the liver to other organs. In the testis, via Sertoli cells, it indirectly delivers Se to spermatic cell lines (particularly to spermatids). Role in steroidogenesis in Leydig cells, where it, induced by gonadotropin stimulation, exerts an anti-oxidative role against lipid (phospholipid and cholesterol) peroxides and peroxynitrite. Possible anti-oxidative role in seminal plasma.	Infertility, reduced number of spermatozoa, abnormal morphology of spermatozoa (tail bending in a hairpin form, mitochondrial swelling, “giant-head” spermatozoa, truncated mitochondrial capsule, lack of mitochondrial capsule, fusion of midpiece and tail, and extrusion of microtubules and outer dense fibers from the tail), decreased motility, decreased sperm DNA viability, and incompletely condensed chromatin. Morphological changes in the testis and epididymis. 80% reduction of Se in the testis. The changes are the same as on the Se-deficient diet or with the ApoER2 knockout.
Selenoprotein V	SELENOV (SelV)	Testis (later stages of spermatogenic cell lines), prostate; cytoplasmic and nuclear localization. Mainly in rodents.	Anti-oxidative role; regulation of other selenoprotein expression in the testis (GPX1, TXNRD3, SELENOP).	Normal fertility, decreased amount of other selenoproteins in testes (GPX4, TRxR3, SELENOP).

SeCys, selenocystein; apoER2, apolipoprotein E receptor 2.

The total amount of Se in the testes is often analyzed by measuring the amount of GPX4 they contain (135). In accordance, the infertile men were shown to have low levels of GPX4 expression and activity in their semen (136), and the lack of GPX4 activity (measured by phosphatidylcholine hydroperoxide consumption) is particularly pronounced in oligoasthenozoospermic men (137). Additionally, the GPX4 activity in semen in that study correlated positively with viability, normal morphology, and particularly progressive motility and maintenance of motility with time (137). While all fertile men had a normal expression of GPX4 in sperm, 10% of the infertile men had a reduced expression of GPX4 (138). In infertile men, low mGPX4 expression in spermatozoa was associated with oligoasthenozoospermia (low sperm count, morphological alterations in spermatozoa, impaired spermatozoa motility, and progressive loss of motility over time) and defective mitochondrial morphology and function (138). Therefore, it seems that GPX4 is the most important for sperm motility and fertilization capacity. During IVF, a lower GPX4 mRNA expression in human sperm was associated only with more asymmetric embryos at day 3, with no effect on the later phase of *in vitro* development (at days 5 and 6) or pregnancy rates (139). In other studies, no association between seminal plasma GPX activity (assessed by the NADPH consumption in the presence of GSH) and IVF outcomes was found, but without specifying the isozyme involved (132, 140). Moreover, in one of them (132), GPX activity was not associated with spermatozoa morphology and motility, but in the other (140), it was linked with lower spermatozoa number, motility, and abnormal morphology. In a separate study, GPX activity in the seminal plasma of infertile men (measured by the NADPH consumption in the presence of GSH) was 10 times lower than in fertile men (133), but in another, only approximately 1.5 times (GPX activity measured by the GSH consumption) (134). Notably, by using specific antibodies for GPX3, it was shown that most of the GPX activity in the former study (133) was attributed to GPX3. This is in agreement that most of the seminal plasma GPX activity derives from seminal vesicles, prostate, and other accessory glands, but not from the testis and epididymis (132), and that > 85% of the semen Se originates from the seminal plasma, and just a small proportion from the spermatozoa (88). As previously mentioned, seminal plasma selenoprotein P concentrations correlated positively with sperm density and vitality in one study (131).

In conclusion, although there is substantial evidence in animals that numerous selenoproteins (e.g., mGPX4, nGPX4, GPX5, TXNRD3, selenoprotein P) are crucial for normal male reproductive function, and that their deficit (genetic knockdown) leads to reduced or total infertility, the studies in humans are still scarce. Generally, only the activity of GPX in seminal plasma was examined in a few studies, but with different methodologies, and without specifying which GPX isoenzyme was analyzed. In general, data show an association with infertility, but not with IVF outcomes. A similar pattern was found for the GPX4 expression in semen. Additionally, the association of genetic polymorphisms of the *gpx4* gene with infertility was shown in some exceptional cases. However, all these studies are quite rare. Regarding selenoprotein P, only one study is available. Therefore, more studies in humans are needed to support the findings in animals, with a unique methodology, specifying the exact proteins/isoenzymes analyzed, with an adequate number of participants involved, a clear distinction of infertile and fertile men from infertile couples, with defined type of infertility (according to seminogram characteristics, confirmed after two semen analyses), careful control of possible confounding, and elimination of other possible known reasons for male infertility.

## 3 Overview of antioxidative and other molecular mechanisms through which Se influences male fertility

As previously mentioned, certain amounts of oxidative stress and ROS are necessary for normal sperm maturation, motility, activation, and fertilizing ability (18, 94, 118, 141, 142). ROS promote formation of disulfide bonds, which are necessary for sperm nucleus compaction and protection, as well as proper mitochondrial structure and function (18, 94, 142–144). Disulfide bonds further protect sperm proteins and DNA from oxidative stress (18). However, there should be a delicate balance between ROS generation and antioxidant defense to avoid an increased oxidative stress, which could be detrimental to sperm (141, 142).

Of all the potential risk factors in idiopathic male infertility, the greatest importance is attributed to increased oxidative stress and altered levels of trace elements (118, 145–147). The presence of oxidative stress in infertile men ranges from 25 to 87% (141). However, the general impact of ROS and reduced antioxidant protection on male infertility is not the subject of this review and can be found elsewhere (41, 146, 147). Spermatozoa are susceptible to oxidative stress due to a lack of their own cellular cytoplasmic antioxidants, high DNA susceptibility to oxidative damage, the presence of abundant polyunsaturated fatty acids (PUFAs) in their membrane, and high ROS production during their movements, generated by mitochondrial activity (18, 85, 89, 118). They rely to a large extent on extracellular antioxidants in epididymal and seminal fluid (18, 118, 141).

As is well known, Se deficiency can reduce selenoprotein biosynthesis, thus disrupting antioxidant defenses in male reproductive organs, and increasing the production of ROS (148). Dietary Se deficiency is associated with increased nucleotide catabolism and enhances radiation-induced micronuclei formation, while treatment with Se protects against DNA deterioration (41, 149).

One of the reliable biomarkers for assessing the degree of lipid peroxidation is the measurement of malondialdehyde (MDA) levels in seminal and blood plasma; MDA is formed when ROS degrade polyunsaturated lipids (150). Interestingly, dietary Se excess is also associated with increased lipid peroxidation, MDA, and ROS accumulation (85).

Additionally, Se can interact with vitamin E and vitamin C, enabling them to scavenge free radicals that can be present among membrane lipids; thus, Se acts synergistically with vitamin E through its role in the GPX system, regenerating vitamin E (in conjunction with vitamin C) and further reducing lipid peroxides (141, 151).

The disulfide bonds are reversible, and they can again be reduced to thiol groups in proteins (e.g., protamine Cys residues) (152). During fertilization, the nucleus again becomes decompacted, and disulfide bonds and protamine oligomerization need to be reversed for the nascent male pronucleus formation. Although this process is poorly understood, it seems that it includes the TXN, TXNRD3, and GRX (e.g., nGPX4), GSH, GPXs, and glutathione S-transferases (GSTOs) systems (153–157).

Not only are spermatozoa affected by Se deficiency, but other cells in the testis are also affected. Dietary Se is also included in the protection of the testis germ cells from oxidative stress, endoplasmic reticulum stress, and apoptosis by modifying extracellular signal-regulated kinase (ERK) and mitogen-activated protein kinase (MAPK) signaling (41, 158). In sheep spermatogonial cells, the effect of Se exhibited the U-shaped response: the optimal medium Se concentrations increased stem cell proliferation and decreased apoptosis by inhibiting ROS production (159). In calves, Sertoli cells, Se improved cell viability and expression of blood–testis-barrier proteins (occludin, connexin-43, zonula occluden, E-cadherin) by modulating the expression of mitochondria-related genes, inhibiting the nuclear factor kappa B (NFκB) activation of inflammatory cytokines and apoptosis, and increasing the GPX4 activity (160, 161). The other cellular mechanisms were also proposed, including modulation of the expression of the transcription factor activator protein 1 (AP1) and the proto-oncogenes cJun and cFos, LH-receptor, and selenoprotein W expression (100, 162). The doses above and below those physiological exerted the opposite effects, possibly through pro-oxidative action of the Se excess (100, 159, 161).

Regarding the effect of Se on testosterone biosynthesis, Se can affect the HPG axis, including both central brain mechanisms and the Leydig cells that produce testosterone (163). Still, there are no data describing a direct effect of Se on GnRH secretion, but indirect effects can be mediated by the effects on the thyroid gland hormone production and amelioration of the hypothyroidism-induced decrease in the brain GnRH secretion and amount (164). Considering LH, Se administration was shown to increase serum LH secretion in mice, and it was also shown to reverse the glucocorticoid-induced decrease in serum LH levels (165). Other researchers have shown increased serum LH production in goats after Se supplementation (166, 167). On the other hand, a more direct effect of Se on testicular testosterone production was proposed by some authors (163). In Se-deficient mice, the testosterone production after GnRH or LH stimulation was reduced; however, the effect of Se deficiency on LH secretion after GnRH stimulation was not observed (168, 169). GPX4 mRNA was also expressed in rat Leydig cells, although much less than in spermatogenic cells (114). In addition, selenoprotein P mRNA was also expressed in rat Leydig cells, and its expression was stimulated during steroidogenesis (170), indicating the possible role in the antioxidant defense against increased ROS production during steroidogenesis (100).

However, associations between Se levels in seminal plasma and blood with testosterone levels have been little studied. Liu et al. (171), examining 1,136 men (mean age: 32.0 ± 6.29 years), found significant dose-dependent correlations between seminal Se levels and dihydrotestosterone (DHT, active form of testosterone), while Akinloye et al. (172) found a significant positive association between serum Se levels and seminal plasma testosterone levels. Similar findings were observed by Oluboyo et al. (173), with a positive correlation between the serum Se and testosterone levels among infertile men. Furthermore, Safarinejad and Safarinejad (174) found a significant increase in testosterone levels after Se supplementation. In animals, Se supplementation increased testosterone levels and enhanced sperm quality (100). *In vitro*, adequate Se concentrations decreased apoptosis and oxidative stress markers and increased proliferation and testosterone production of sheep Leydig cells by affecting p-ERK1/2 signaling, steroidogenic enzyme activities, and miR-200a/NRF antioxidant pathway (168). Interestingly, maternal dietary Se supplementation during gestation and lactation in goats increased testicular weight and volume, density of spermatogenic and Leydig cells, expression of enzymes in testosterone biosynthesis, androgenic receptor protein, and levels of testosterone in testicular tissue and serum (175). Similar effects were observed in calves (176).

An additional mechanism by which Se can modulate the reproductive function in men is through its influence on thyroid gland hormone production. Thyroid hormones are essential for the normal function of the HPG axis, including Sertoli, Leydig, and spermatic cells in testes, sperm count, morphology, motility, and fertilizing ability, the morphology and function of epididymis, seminal vesicle, and prostate, and regulation of oxidative stress (25, 177), and their imbalance by Se deficiency/excess can be referred to reproductive function (177–179). The same stands for insulin secretion and insulin sensitivity, which are also very important for normal reproductive function (22, 180), but can be disturbed by Se deficiency or excess (181).

Overall, there is substantial evidence that Se can significantly influence male reproductive function through various mechanisms involving selenoproteins, including both direct and indirect effects on the HPG axis.

## 4 Effect of Se deficiency, substitution, and excess on male fertility in experimental and domestic animals and humans

Experiments with Se-deficient diets in animals have shown reduced motility and poorer sperm quality compared to diets with higher Se content (135). The best-characterized effects of Se deficiency on sperm include significant loss of motility, midpiece breakage, and increased sperm shape abnormalities (mostly with an abnormal sperm head or bent tail, “hairpin-like tail,” and an increased percentage of tailless and headless sperms) (85). Consequently, Se deficiency can lead to inadequate chromatin condensation, which, in turn, affects sperm quality and causes reduced fertilization capacity (182). In mice fed Se deficient diets, testicular atrophy, reduced diameter of the seminiferous tubules, osseous metaplasia, and reduced spermatogenic activity were observed: no mitotic activity in spermatogonia, reduced the number of spermatogenic cell lines (including stem cells, pachytene spermatocytes, spermatids, and maturing sperms), and higher rates of abnormal morphology of sperm were found without difference in chromosomal abnormalities in spermatocytes (85, 100). These testicular morphological changes were more pronounced compared to changes observed in mice lacking mGPX (103). In the epididymis of Se-deficient rats, more undeveloped and degenerated spermatozoa were found, with decreased motility (169). The lack of MCP in spermatozoa was also described, associated with abnormal morphology of the mitochondrial capsules, with gaps between consecutive mitochondria, mitochondrial swelling, mitochondrial loss, cytoplasmic droplets, loose contacts of the mitochondrial helix with the plasma membrane, separations of the flagellar fibers and their protrusion, partially condensed chromatin, and fusions of midpiece and tail (104, 105, 169). Interestingly, the next generations of mice had more profound defects (85, 169). In boars fed a Se-deficient diet (corn and soybeans grown in the Se-deficient areas of China), reduced sperm number and viability, and morphological defects in heads, midpiece, tail, plasma membrane, and mitochondrial gaps were described (85, 169). In roosters, a Se-deficient diet also led to a high percentage of abnormal spermatozoa (85). However, most data indicate that only extremely low Se intake influences male livestock fertility (169). Overall, studies in experimental and domestic animals on Se-deficient diets indicate poorer semen and sperm quality, as well as morphological changes in the testes and accessory organs, resulting in decreased fertility, particularly with significant deficits.

In contrast, studies in animals fed a feed supplemented with Se (both livestock and experimental animals) have shown better sperm characteristics and fertilizing ability (169, 183). Selenium supplementation also increased seminal plasma and epididymal fluid production by stimulating the development of primary and secondary glands (183). Studies in rams showed that Se supplementation increased testosterone levels, sperm motility, volume, and concentration, decreased the percentage of abnormal sperm, dead sperm, or sperm with acrosome damage, and increased scrotal circumference (183). Similarly, in male goats, Se supplementation of feed resulted in a significant increase in testosterone and LH secretion, improved volume and morphology of testes, improved ejaculate volume, sperm morphology, activity, and progressive motility (85, 166). Addition of Se to feed in roosters enhanced sexual maturation and semen production, with upregulation of genes involved in testicular morphology and function, increased number of seminiferous tubule cells, viability of Sertoli cells, spermatozoa motility, viability, and percent normal spermatozoa, particularly after organic Se substitution (85). In boars, Se supplementation with inorganic Se improved sperm motility and decreased the percentage of abnormal spermatozoa, with increasing fertilization rates and increased ejaculate volume, but decreased sperm concentration, while organic Se supplementation increased sperm concentration and total number of sperm per ejaculate and seminal doses (85, 184). In addition, an increased libido in supplemented rams and boars was shown (85).

Regarding humans, unfortunately, fertility studies on patients with Kashin–Beck and Keshan diseases, i.e., severe Se deficiency, have never been conducted. Interestingly, in healthy men, low dietary Se intake (13 μg/day for ~100 days) decreased serum, erythrocyte, and seminal plasma (but not sperm) Se concentrations, without influencing serum androgen, LH, and FSH concentrations, and decreased concentration and total number of sperm, increased the proportion of tailless sperm, but decreased the proportion of headless sperm (185). Unfortunately, the number of subjects included in this study was only six. In contrast, in infertile men, Se treatment (200 μg Se per day for 3 months in the form of high-Se yeast) improved sperm motility (but not concentration), together with improved anti-oxidant status, total antioxidant capacity (TAC), and superoxide dismutase (SOD) activity (186). In another study in infertile men, Se treatment (l-selenomethionine 100 μg per day for 3 months) also increased blood plasma Se concentrations and sperm motility without affecting sperm density (187).

However, also dietary/drinking water Se excess is associated with infertility and testicular degeneration in experimental animals, related to higher GPX4 and lactate dehydrogenase activity and higher hydrogen selenide production, and reduced spermatozoa motility, concentration, and typical spermatozoa morphological defects (with the fusion of two midpieces, vacuolization of the cytoplasm, and twisted tails) (85, 105, 117, 169). Also in livestock (boars), retarded sexual development was shown with Se excess in feed, with a gradual decline in sperm production (85, 169). Unfortunately, studies on fertility in regions with endemic selenosis have also not been conducted. In six healthy (free-living, fertile) US men, a diet with a high Se intake (~200 μg/day for ~100 days, mostly through Se-rich beef and rice) decreased concentration and total number of sperm, the proportion of motile, progressive, headless sperm, and tail defects, and increased the proportion of tailless sperm (185). However, in another study in 42 healthy US men, Se supplementation (300 μg per day as high-Se yeast for 48 weeks) increased serum and seminal plasma Se concentrations but did not affect sperm Se concentration, serum androgen concentrations, or semen quality (sperm count, motility, progressive velocity, or morphology) (188) (More on the effects of Se supplementation on human sperm quality will be discussed in Section 5.4).

Overall, both Se deficiency and excess can lead to reduced semen quality and fertility in males. This is supported by evidence from animal studies, but there is less research available in humans. Nevertheless, the experimental conditions in animals—characterized by severe dietary deficiencies, excesses, or genetic knockdowns—result in more pronounced Se imbalances within the reproductive system. Consequently, these imbalances lead to more significant changes and consequences compared to those usually observed in humans or domestic animals. Therefore, more studies are needed in humans to establish the optimal levels of Se intake and Se biomarkers in the body associated with the optimal fertility parameters.

## 5 Review of available human studies on the association between seminal plasma Se levels and male fertility, semen quality, and the effects of Se supplementation on semen quality

In the following sections, we provide an overview of the published data regarding the human seminal plasma Se levels in fertile/normospermic men compared to infertile/oligozoospermic/azoospermic men worldwide and associations with semen quality parameters. We will also discuss findings from studies on the effects of Se supplementation on semen quality parameters. This part of the manuscript will focus exclusively on Se concentrations in seminal plasma, due to the sufficient amount of available data. Data on measurements of GPX levels and activity in seminal plasma and sperm cells, as well as selenoprotein Se concentrations, and their associations with the seminogram quality are not included in this section, as the available data are limited and these topics have already been discussed in Section 3. Additionally, it is important to note that there is a deficit of studies on selenoprotein P concentrations in human seminal plasma, and there is considerable heterogeneity in the measurement of GPX concentrations and activity, which is a limiting factor for making comparisons and reaching conclusions.

### 5.1 Seminal plasma Se levels worldwide in fertile/normospermic/healthy men

**Figure 2:**
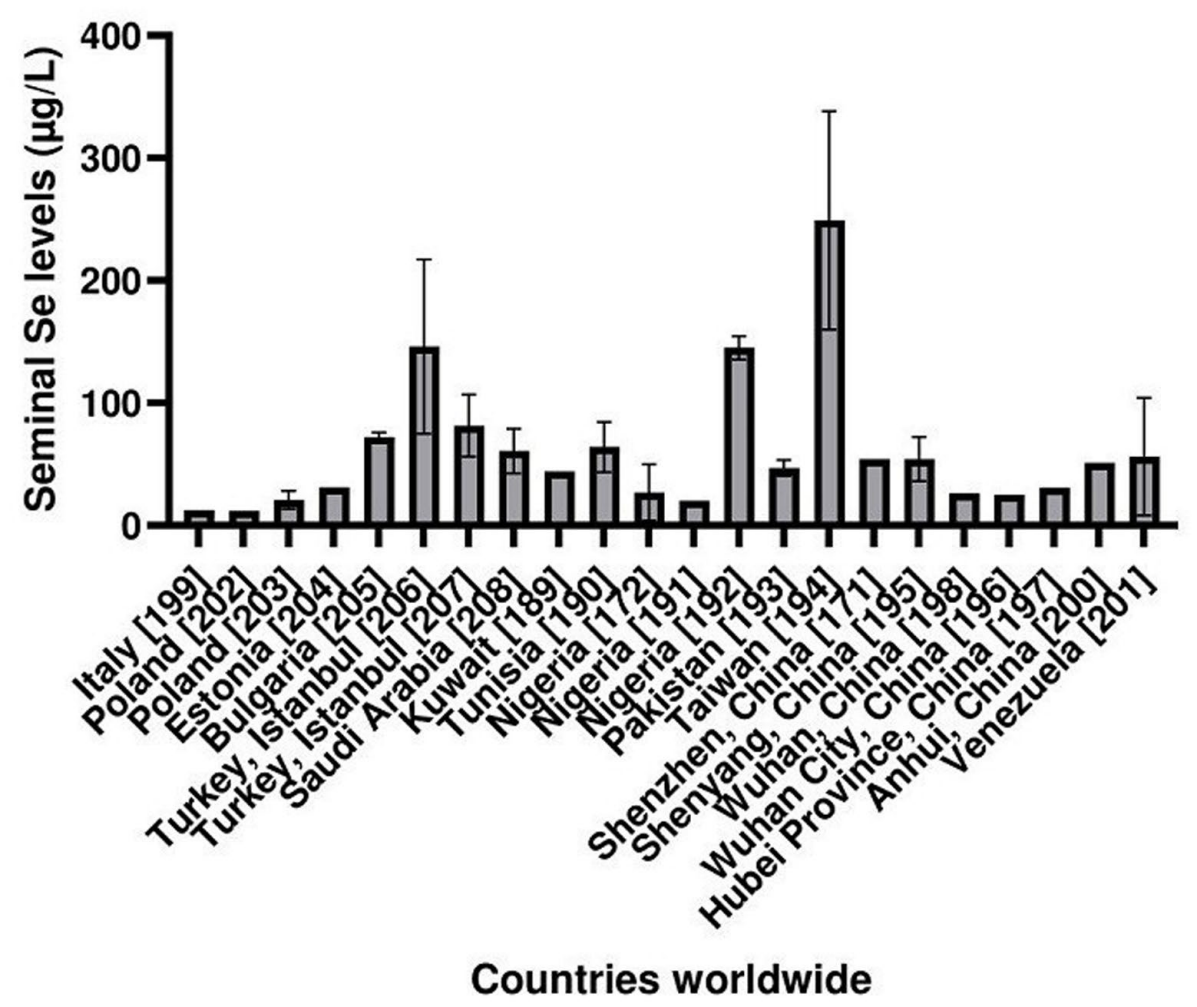
Selenium levels in the seminal plasma of fertile men in countries or regions around the world (171, 172, 189–208). Data are shown as mean and standard deviation (upper cap) or as median (μg/L).

According to the literature search, Se levels in the seminal plasma of fertile men are partially available for countries across the globe. In this review, we selected those studies that adhered to the 2010 WHO laboratory manual for the examination and processing of human semen. Moreover, all studies that did not specify the inclusion/exclusion criteria for fertile participants were excluded from this review analysis. The results for Se levels in the seminal plasma of fertile (normospermic) men worldwide are summarized in Figure 2. In general, it can be concluded that Se levels in seminal plasma are not uniform throughout the world (171, 172, 189–208). Moreover, population analysis has shown that Se levels in the seminal plasma of fertile men were much higher in one study from Turkey (207), one study from Nigeria (172), and the study from Taiwan (194) than in other countries. The explanations for these findings are possibly specificities of the geographical region or the analytical method used. For example, in Nigeria, the plasma Se levels varied from as low as 6 μg/L to as high as 328 μg/L, depending on the geographical factors and analytical methods employed (209). In accordance, results in seminal plasma varied from 20 μg/L to 146.1 μg/L in these three studies (172, 191, 192). The high values from one study in Turkey (207) are surprising, considering generally low blood serum/plasma levels of Se in the Turkish population (210). However, in another study, the levels were significantly lower (206). In Taiwan, in one study, including 2,755 subjects, the average serum Se concentrations ranged from 41 to 186 μg/L, on average 111 μg/L (211), but in another, more recent study, including 5,508 subjects, much higher values were reported, ranging from 58 to 307 μg/L, on average 183 μg/L (212). Nevertheless, such high Se concentrations in the seminal plasma (~250 μg/L) in the study from Taiwan (194) are very unusual and unexplained. In particular, in that study (194), the maximal Se concentration was 610 μg/L. There could be a possible influence of the different analytical methods used in that study (electrothermal atomic absorption spectrometry). The lowest Se levels in seminal plasma have been reported for Italy, Poland, Estonia, and specific Chinese regions, primarily Hubei Province (including Wuhan); however, Se levels from other regions of China (Shenzhen, Anhui, Shenyang) were much higher, which is in line with the soil Se content in these regions (56, 57, 213). Thus, the authors of this review encourage researchers to conduct new studies of seminal plasma Se levels in as many countries as possible, by applying the standardized procedures and techniques to make comparable results, to gain a clearer insight into Se status in the fertile male population. It would be particularly interesting to compare seminal plasma Se levels in regions with endemic selenium deficiency and selenosis, alongside a comparison of semen characteristics.

### 5.2 Comparative analysis of seminal and blood Se levels between fertile and infertile men

In this section, we selected those studies that provided Se levels in seminal plasma and blood (serum/plasma) samples of fertile and infertile men analyzed with adequate methodology. Studies with inadequate methodology or insufficient numerical data, studies with subjects of atypical age, and studies in a language other than English were not considered in our paper. The findings are summarized in Table 2.

**Table 2:** Literature data on the comparison of seminal plasma Se levels between men with infertility, abnormal seminogram, or inflammation, and fertile/normozoospermic/healthy men.

References	Country	Seminal plasma Se		Blood/Serum Se	
		Men included in the study and seminal plasma Se values: Mean ± SD or median (IQR)	Significance of difference	Blood/Serum Se values: Mean ± SD or median (IQR)	Significance of difference
Akinloye et al. (172)	Nigeria	100 men from infertile couples (idiopathic): 20 azoospermic men: 245.6 ± 5.5 μg/L; 40 oligozoospermic men: 82.1 ± 7.1 μg/L; 40 age-matched normozoospermic men: 146.1 ± 9.5 μg/L; (proven fertility)	Higher in normozoospermic vs. oligozoospermic men. Higher in azoospermic vs. oligozoospermic and normozoospermic men. Not controlled for confounding	Serum; Azoospermic: 127.9 ± 7.9 μg/L; Oligozoospermic: 330.8 ± 15.0 μg/L; Normozoospermic: 138.2 ± 12.6 μg/L;	Higher in oligozoospermic vs. azoospermic and normozoospermic men
Eroglu et al. (206)	Turkey	59 men from infertile couples (idiopathic): 44 oligozoospermic men: 33.0 ± 18.2 μg/L; 15 age-matched normozoospermic men: 81.4 ± 25.4 μg/L; (proven fertility)	Higher in normozoospermic vs. oligozoospermic men. Controlled for age.	Serum; Oligozoospermic: 81.9 ± 36.3 μg/L; Normozoospermic: 138.1 ± 12.3 μg/L	Higher in normozoospermic vs. oligozoospermic men. Serum Se levels positively correlated with seminal Se levels.
Karabulut et al. (207)	Turkey	113 men from infertile couples (idiopathic): 53 men with at least one abnormal semen parameter (ASP): 106.6 ± 77.0 μg/L; 60 men with normal sperm parameters (NSP): 146.2 ± 71.3 μg/L	Higher in men with NSP vs. ASP. Not controlled for confounding.	/	/
Calogero et al. (199)	Italy	179 men living in rural or industrial areas: 131 men with at least one abnormal semen parameter (ASP): 1.2 (1.2–14.0) μg/L; 48 men with normal sperm parameters (NSP): 12.4 (1.2–35.0) μg/L	Higher in men with NSP vs. ASP. Controlled for age. Other confounding factors (environmental, lifestyle) were not matched.	Full blood; ASP: 111.2 (76.3–145.2) μg/L; NSP: 115.2 (80.4–130.8) μg/L	No significant difference. Full blood Se levels negatively correlated with seminal Se levels.
Wdowiak et al. (203)	Poland	131 infertile men (idiopathic): mean 8.8 μg/L; 85 proven fertile men: mean 11.8 μg/L	Higher in fertile vs. infertile men. Not controlled for age and other confounders. Underweight and overweight individuals were excluded.	/	/
Aljaser et al. (208)	Saudi Arabia	70 men from infertile couples (idiopathic): 30 asthenozoospermic men: 43.1 ± 19.7 μg/L; 40 age-matched normozoospermic men: 60.7 ± 18.1 μg/L; proven fertility	Higher in normozoospermic men, but no significant difference. Controlled only for age.	/	/
Atig et al. (190)	Tunisia	190 men from infertile couples (idiopathic): 74 asthenozoospermic men: 56.0 ± 22.8 μg/L; 56 oligozoospermic men: 55.0 ± 21.4 μg/L; 60 teratozoospermic men: 57.0 ± 24.3 μg/L; 60 normozoospermic men: 64.0 ± 20.6 μg/L; proven fertility	Higher in normozoospermic men, but no significant difference. Controlled for age, but normozoospermic and astenospermic were non-significantly younger.	/	/
Jeng et al. (194)	Taiwan	196 generally healthy men on a regular health screening: Sperm concentration: ≥15 × 10^6^/ml: 122.6 ± 128.0 μg/L; <15 × 10^6^/ml: 179.1 ± 140.3 μg/L; Motility: ≥40%: 96.1 ± 151.0 μg/L; <40%: 133.1 ± 136.4 μg/L; Viability: ≥58%: 101.1 ± 113.2 μg/L; <58%: 140.1 ± 133.7 μg/L Normal morphology: ≥4%: 110.3 ± 142.9 μg/L; <4%: 138.9 ± 136.5 μg/L;	No significant difference (huge variability). Controlled for main confounders	/	/
Yin et al. (214)	China	148 men from infertile couples (idiopathic): 73 oligoasthenoteratozoospermic men: geometric mean 74.3 μg/L; 75 normozoospermic men: geometric mean 78.0 μg/L;	Higher in normozoospermic men, but no significant difference. Controlled for main confounders.	/	/
Chinyere Nsonwu-Anyanwu et al. (192)	Nigeria	130 men from infertile couples (idiopathic): 30 azoospermic men: 20.9 ± 10,0 μg/L; 50 oligozoospermic men: 23.8 ± 12.6 μg/L; 50 normozoospermic men: 26.7 ± 22.9 μg/L;	Higher in normozoospermic men, but no significant difference. Controlled for age and BMI.	/	/
Bassey et al. (191)	Nigeria	80 men with idiopathic infertility 20 azoospermic men: 19.0 ± 0.1 μg/L; 18 oligozoospermic men: 19.1 ± 0.1 μg/L; 42 oligoasthenozoospermic men: 20.2 ± 0.1 μg/L; 62 normozoospermic men: 20.0 ± 0.1 μg/L; proven fertility	No significant difference. Controlled for age.	/	/
Camejo et al. (201)	Venezuela	67 men with varicocele grade II + III: 38.2 ± 36.4 μg/L; 44 normozoospermic men: 56.1 ± 48.1 μg/L	Higher in normozoospermic men vs. men with varicocele, controlled for age.	/	/
Türk et al. (204)	Estonia	29 men with severe inflammation in semen: 3.5 (1.5–11.0) μg/L; 31 men with severe inflammation in expressed prostatic secretion or post-massage urine: 3.2 (1.5–6.2) μg/L; 24 men with mild inflammation in semen, expressed prostatic secretion, or post-massage urine: 4.2 (2.4–11.6) μg/L; 32 men with non-inflammatory oligozoospermia: 11.0 (5.8–14.5) μg/L; 27 healthy men: 31.0 (25.0–36.0) μg/L; proven fertility	Higher in healthy men vs. men with inflammation and men with non-inflammatory oligozoospermia. Controlled for age.	/	/
Wu et al. (195)	China	103 men from infertile couples, with infertility: 33.0 ± 4.8 μg/L	A positive association between seminal Se levels and the likelihood of pregnancy and live birth	/	/

SD, standard deviation; IQR, interquartile range; ASP, abnormal semen parameters; NSP, normal sperm parameters; BMI, body mass index.

Overall, some studies did not find significant differences (six studies) (190–192, 194, 208, 214), but some showed lower seminal plasma Se levels in infertile men, men with abnormal sperm parameters, or inflammation, compared to fertile/normozoospermic/healthy men (five studies) (172, 199, 203, 206, 207). However, in four studies, there were observed higher Se levels were observed in infertile/normozoospermic/healthy men, although statistical significance was not observed (190, 192, 208, 214), probably due to a low number of the included subjects. Another explanation is that there could be a U-shaped association between seminal plasma Se levels and infertility. In agreement, in one study (172), significantly higher seminal plasma Se levels in azoospermic men were found, indicating that Se excess in seminal plasma can also be associated with infertility, not only Se insufficient levels. In one study (195), a positive association of seminal Se levels with the likelihood of pregnancy during IVF and live birth was found. In general, studies included a low to moderate number of participants, but were either not controlled for confounding or were controlled only for age, rarely for other important variables. Additionally, different techniques were included for Se determination.

### 5.3 Associations between Se levels in seminal plasma and blood with sperm quality

Data from the literature on the associations of Se levels in seminal plasma with seminogram parameters are summarized in Table 3, first as case–control studies (seven studies) and second as cross-sectional studies (five studies). Overall, these data allow the conclusion that Se levels in seminal plasma and/or serum are positively associated with good seminogram parameters, with the strongest association observed with sperm motility (as shown in 10 of 13 studies included) (171, 190, 200–202, 204, 206, 208, 215). Only one study (172) observed negative correlations of serum and seminal plasma Se levels with sperm count and viability, respectively, but still a positive correlation of seminal plasma Se levels with sperm motility, while two studies observed no correlation (194, 195). Case–control studies were controlled for age (almost all), but rarely also for body weight and smoking, and correlation analyses were applied to the total sample. Many studies (particularly case–control) included only a limited number of subjects, and studies applied various techniques for seminal plasma Se analysis. In general, data are in alignment with results from animal studies, showing that a deficit of Se and selenoproteins leads mainly to lower sperm motility, malformations in sperm midpiece, tail, mitochondria, and flagellar structure (described in Sections 2 and 4).

**Table 3:** Literature data on the influences of Se on seminogram findings in humans.

Reference	Study design	Country	Sample size	Age (years)	Clinical sample	Analytical technique	Simplified main findings
Akinloye et al. (172)	Case–control	Nigeria	40 normozoospermic men and 60 infertile men (40 oligozoospermic and 20 azoospermic)	20–45	Seminal plasma, serum	AAS	A significant negative correlation between serum Se level and sperm count. A significant negative correlation between seminal Se level and sperm vitality. A significant positive correlation between seminal Se level and sperm motility. Data refer to all subjects merged.
Aljaser et al. (208)	Case–control	Saudi Arabia	40 normozoospermic and 30 asthenozoospermic men	36.8 ± 4.9; 37.7 ± 5.4	Seminal plasma	AAS	Significant positive correlations between seminal Se levels and total motility and normal sperm morphology. Data refer to all subjects merged
Atig et al. (190)	Case–control	Tunisia	60 normozoospermic, 74 asthenozoospermic, 56 oligozoospermic, and 60 teratozoospermic men	33.4 ± 4.4; 34.0 ± 4.2; 40.3 ± 4.5; 39.0 ± 5.0	Seminal plasma	AAS	A significant positive correlation between seminal Se level and sperm motility. Data refer to all subjects merged
Camejo et al. (201)	Case–control	Venezuela	44 normozoospermic and 67 men with varicocele	34.3 ± 6.4; 33.6 ± 9.6;	Seminal plasma	TXRF	Significant positive correlations between seminal Se levels and sperm concentrations, motility, and morphology. Data refer to all subjects merged
Chyra-Jach et al. (202)	Case–control	Poland	103 normozoospermic, 384 men with abnormalities in spermogram: 152 oligozoospermic, 142 asthenozoospermic, and 90 oligoasthenozoospermic	33 ± 6; 34 ± 5;	Seminal plasma	AAS	Significant positive correlations between seminal Se levels and sperm count, total sperm count, and sperm motility. Data refer to all subjects merged
Eroglu et al. (206)	Case–control	Turkey	15 normozoospermic and 44 oligozoospermic men	32.5 ± 5.3; 31.2 ± 4.9	Seminal plasma, serum	AAS	Significant positive correlations of serum and seminal Se levels with sperm concentration, motility, and morphology. Data refer to all subjects merged.
Türk et al. (204)	Case–control	Estonia	27 fertile men. 32 men with oligozoospermia, 84 infertile men with inflammatory status: 29 with severe in semen, 31 with severe in prostate, 24 with mild in semen/prostate	31 (25–33); 31(29–35); 31 (28–39); 30 (28–34); 31.5(27–34);	Seminal plasma	ICP-OES	Significant positive correlations between seminal Se levels and sperm count and motility. Data refer to all subjects merged.
Jeng et al. (194)	Cross-sectional	Taiwan	196 free-living men	38.4 ± 9.9	Seminal plasma, urine	AAS	No significant correlations were found between seminal or urine Se levels with sperm quality.
Liu et al. (171)	Cross-sectional	China	1,136 free-living men	32.0 ± 6.0	Seminal plasma	ICP-MS	Significant positive correlations between seminal Se levels and sperm concentration and total sperm count.
Xu et al. (200)	Cross-sectional	China	56 free-living, non-smoking men	34.5 ± 4.4	Seminal plasma	AAS	Significant positive correlations between seminal Se levels and sperm density, sperm number, sperm motility, and sperm viability.
Chen et al. (197)	Cross-sectional	China	1,159 potential sperm donors	28.1 ± 5.3	Seminal plasma	ICP-MS	Significant positive correlations between seminal Se levels and sperm concentration and total count.
Talebi et al. (215)	Cross-sectional	Iran	350 men attending the IVF clinic	34.8 ± 0.4	Seminal plasma	ICP-MS	Significant positive correlations between seminal Se levels and semen volume and sperm motility.
Wu et al. (195)	Cross-sectional	China	103 men attending the IVF clinic, with idiopathic infertility	33.0 ± 4.8	Seminal plasma	ICP-MS	No significant associations were found between seminal Se levels with sperm quality.

N.P., Not Provided; IVF, in vitro fertilization; AAS, Atomic Absorption Spectroscopy; TXRF, Total Reflection X-ray Fluorescence; ICP-OES, Inductively Coupled Plasma-Optical Emission Spectroscopy; ICP-MS, Inductively Coupled Plasma-Mass Spectrometry.

### 5.4 Effects of Se supplementation on semen quality

The effects of Se supplementation therapy on semen parameters have been modestly investigated over the last 35 years and are shown in Table 4. Note: studies using other supplements combined with Se were also included in this overview, but separately analyzed.

**Table 4:** Effects of Se supplementation therapy on semen parameters in humans.

Reference	Type of study	Number of subjects in total	Age (years)	Characteristics of men included in the study	Basal Se levels in the Se-treatment group	Basal sperm motility (%)	Treatment	Time of treatment (months)	Main findings
Dietary interventions									
Hawkes and Turek (185)	Double blind RCT, dietary	12 (11 finished)	20–45	Healthy men from the USA (California)	Seminal plasma: 43 ± 11 μg/L Blood plasma: 120 ± 20 μg/L	5.88 ± 1.61	Dietary intervention: First 21 days: a control baseline diet, with dietary Se 47 μg/d. Next, 99 days: Dietary Se 297 μg/d (*n* = 6, high Se diet, through meat and rice high in Se) Or Dietary Se 13 μg/d (*n* = 6, low Se diet, through meat and rice low in Se)	4	Reduced fraction of motile sperm in the high-Se group.
Se-only supplements									
Iwainer et al. (219)	Two-group intervention study, no control group	33	19–38 (mean 31)	Subfertile men from Poland	Seminal plasma: 28.0 ± 9.5 μg/L Blood plasma: 62.4 ± 8.8 μg/L Whole blood: 80.4 ± 9.7 μg/L	30.5	200 μg/d of Se-rich-yeast (*n* = 16) Or 200 μg/d of Na-selenite + baker’s yeast (n = 17)	3	No effect on sperm motility, count, or morphology in both groups.
Scott et al. (187)	Double blind RCT	64	33.3 ± 0.6	Men with asthenozoospermia from the UK (Scotland)	Blood plasma: 82.5 ± 3.3 μg/L	21.6 ± 4,1	100 μg/d L-SeMet (*n* = 16) Or 100 μg/d SeMet + vitamins A (1 mg), C (10 mg), E (15 mg) (*n* = 30) Or Placebo (*n* = 18)	3	Positive effects on sperm motility at the borderline of statistical significance, but no effect on sperm count. No additional effect of vitamins
Hawkes et al. (188)	Double blind RCT	54 (42 completed)	18–45	Healthy men from the USA (California)	Seminal plasma: 45.0 ± 13.4 μg/L Blood plasma: 142.1 ± 19.0 μg/L	67 ± 17	300 μg/d of Se yeast (*n* = 22) Or Placebo yeast (*n* = 20)	12	No effect on sperm motility, count, or morphology.
Safarinejad and Safarinejad (174)	Double blind RCT	468 (420 completed)	25–48	Men with idiopathic OAT from Iran	Seminal plasma: 22.6 ± 4.4 μg/L Blood plasma: 77.7 ± 6.8 μg/L	22.4 ± 5.2	200 μg/d of Se (*n* = 105) Or 600 mg/d of NAC (*n* = 105) Or 200 μg/d of Se + 600 mg/d of NAC (*n* = 104) Or Placebo (*n* = 106)	6	Positive effects on sperm motility, count, and morphology. Additional effect of NAC.
Alahmar and Sengupta (186)	RCT	70	25.4 ± 7.7	Men with idiopathic OAT from Iraq	N.P.	28.7 ± 5.4	200 μg/d of Se (*n* = 35) Or 200 mg of CoQ10 (*n* = 35)	3	Positive effects on sperm concentration, progressive motility, and total motility.
Alahmar (217)	Single-group intervention study	65	35.2 ± 12.5	Men with idiopathic OAT from Iraq	N.P.	29.3 ± 8.5	200 μg/d of L-SeMet	6	Positive effects on sperm concentration, progressive motility, total motility, sperm DNA fragmentation, and seminal plasma antioxidant capacity
Alahmar (218)	Two-group intervention study, no control group	80	34.3 ± 9.2 and 32.4 ± 11.5	Men with idiopathic OAT (40) and varicocele (40) from Iraq	N.P.	23 ± 6.2 and 21.6 ± 6.9	200 μg/d of L-SeMet in both groups	3	Positive effects on progressive motility and total motility in both groups, but higher in men with varicocele.
Cannarella et al. (216)	Single-group intervention study, no control group	20	32.2 ± 7.1	Men with autoimmune thyroiditis	N.P.	N.P.	83 μg/d of Se yeast	6	Positive effects on sperm concentration, progressive motility, and morphology. Lower percentage of spermatozoa with DNA fragmentation and lower leukocyte concentration in the seminal plasma
Meta-analyses									
Salas-Huetos et al. (220)	Meta-analysis of three RCTs: Hawkes et al. (188), Safarinejad and Safarinejad (174), Scott et al. (187)	287	N.P.	42 healthy men (188), 211 men with idiopathic OAT (174), 34 men with asthenozoospermia (187)	N.P.	N.P.	100–300 μg Se/d	3–11	Positive effects on sperm concentration, total motility, and morphology
Buhling et al. (221)	Meta-analysis of two RCTs: Safarinejad and Safarinejad (174), Scott et al. (187)	245	N.P.	211 men with idiopathic OAT (174), 34 men with idiopathic OAT (187)	N.P.	N.P.	100–200 μg Se/d	3–6	Positive effects on sperm concentration, total motility
Li et al. (222)	Network meta-analysis of two RCTs: Safarinejad and Safarinejad (174), Scott et al. (187)	245	N.P.	211 men with idiopathic OAT (174), 34 men with idiopathic OAT (187)	N.P.	N.P.	100–200 μg Se/d	3–6	Positive effects on sperm concentrations, total motility, and morphology in pairwise meta-analysis, but in network meta-analysis, the effect was lost.
Su et al. (223)	Network meta-analysis of two RCTs: Safarinejad and Safarinejad (174), Scott et al. (187)	245	N.P.	211 men with idiopathic OAT (174), 34 men with idiopathic OAT (187)	N.P.	N.P.	100–200 μg Se/d	3–6	Positive effects on sperm concentration and morphology in pairwise meta-analysis, but in network meta-analysis, the effect was lost.
Se combined with other supplements									
Vezina et al. (224)	Single-group intervention study, no control group	9	28–36	Men with OAT from Canada (Québec)	Seminal plasma: 68.3 μg/L	12.1	1st month: 100 μg/d organic Se + 400 mg/d vit E 2nd–6th month: 200 μg/d organic Se + 400 mg/d vit E	6	Positive effects on sperm motility, vitality, and morphology.
Keskes-Ammar et al. (225)	Open-label RCT	54	N. P.	Healthy and infertile men from Tunisia	N.P.	34	225 μg Se + 400 mg vit E (*n* = 28) Or 4.5 g/d vit B (*n* = 26)	3	Positive effects on sperm motility.
Moslemi and Tavanbakhsh (226)	Single-group intervention study, no control group	690	20–45	Men with idiopathic asthenoteratozoospermia from Iran	N.P.	10–30	200 μg/d of SeMet + 400 IU/d vit E	3	Positive effects on sperm motility, morphology, and pregnancy rate.
Lombardo et al. (227)	Single-group intervention study, no control group	60	30–55	Men with chronic prostatitis from Italy	N.P.	18	82.3 μg Se + 1.5 mg lycopene + 250 mg epigallocatechin gallate + 250 mg ellagic acid + 20 mg Zn (*n* = 30) Or Without treatment (*n* = 30)	6	Positive effects on sperm motility and a drop in atypical morphology.
Sabeti et al. (228)	Single-blind RCT	60	31.9 ± 3.7	Men with asthenoteratozoospermia from Iran	N.P.	28.8 ± 6.7	200 μg of Se + 400 IU of vit E (*n* = 30) Or Placebo (*n* = 30)	3	Positive effects on sperm motility and viability.
Bahmiary et al. (229)	Single-blind RCT	62	18–55 (37.2 ± 7.1)	Men with idiopathic OAT from Iran	N.P.	25.6 ± 17.1	200 μg of Se + 400 IU of vit E + 5 mg folic acid (*n* = 30) Or Placebo (*n* = 32)	3	Positive effects on sperm motility index and functional sperm concentration, but with no difference compared to placebo.
Rochdi et al. (230)	Single-group intervention study, no control group	420	26–59 (38.5 ± 1.2)	Men with idiopathic OAT from Morocco	N.P.	28.5 ± 1.0	50 μg of Se + 80 mg Vitamin C + 0.2 mg Vitamin B9 + 100 mg Zinc + 400 mg Arginine + 400 mg L-carnitine + 30 mg Coenzyme Q10	6	Positive effects on sperm concentration, progressive, and total motility. Lower percentage of spermatozoa with DNA fragmentation, non-significantly increased FSH, and testosterone.

RCT, Randomized Controlled Trial; OAT, Oligoasthenoteratozoospermia; N.P., Not Provided; SeMet, Selenomethionine; NAC, N-Acetyl Cysteine; Zn, Zinc; n, number of subjects enrolled.

There was a high variability among the obtained results from the Se-only supplementation, but most studies (five of seven studies) have found positive effects, particularly on sperm motility (174, 186, 187, 216–218), and in some, also sperm concentration and/or morphology (174, 186, 216, 217). Some studies, however, have not found the effect (188, 219). Rare meta-analyses performed (only four), with only up to three studies included in the analyses, also confirmed positive effects (but only in pairwise meta-analyses, not in network meta-analyses) (220–223). However, the limited number of studies included in these meta-analyses limits their authority. One study, which found negative effects on sperm motility, was a Se dietary intervention, but in only six healthy subjects, with normal Se intake and serum levels (185). The findings from Se-only supplementation studies were in accordance with the studies using the other supplements combined with Se (e.g., vitamin E, or vitamin E + folic acid, or Zn + lycopene + epigallocatechin gallate + ellagic acid, or vitamin C + vitamin E + Zn + arginine + L-carnitine + coenzyme Q10), which also confirmed positive effects in six of seven studies (224–230). Nevertheless, the effects of Se in them cannot be distinguished from the effects of other supplements.

The variability in results could be due to differences in Se supplement form (diet or supplement; organic or inorganic), dosage (daily intake), duration of intervention, inclusion criteria (healthy men or men with various types of infertility), exclusion criteria, and baseline Se levels, which likely vary by geographic region. In the Se-only supplementation studies, just one study included more subjects (420), while others included only up to 20–80 subjects in total. The number of participants in the Se-only arm varied from 16 to 105 subjects. Some were randomized controlled trials (RCT, double-blinded or no-blinded), some were one-group or two-group intervention studies with Se, without a control group, or controlled with another treatment. The control for possible confounding (e.g., age, obesity, smoking) generally was not performed (only in one study). As already mentioned, in studies with Se combined with other supplements, the effect of Se cannot be distinguished, which makes them not suitable for a possible meta-analysis.

Selenium supplementation appears to have a greater impact on motility (particularly progressive motility) than on other semen parameters; significant improvement was observed in cases with notably reduced sperm motility at baseline (≤ 20%). Above these values, Se supplementation is questionable (90). After cessation of Se supplementation, parameters returned to baseline in about two spermatogenesis cycles (26 weeks) (174). The effect on motility is in accordance with the studies in animals (both dietary or selenoproteins’ knockout interventions), which have shown defects in mitochondria, midpiece, and tails of spermatozoa, resulting in lower motility, viability, and fertilizing ability (see Sections 2 and 4).

The beneficial effect of Se supplementation also depends on the dose (51, 231). Selenium supplement dosage should be carefully considered, especially in the USA, where baseline Se intake is generally higher. In California, some parts have excessive Se contents in soil, water, plants, and animals (232). At a Se intake of 70 μg/day, the predicted plasma Se concentrations would be ~ 100–110 μg/L (51), and by taking 200 μg/day of Se, roughly all adults would have plasma Se levels above 100 μg/L after several months. For example, in the studies (174, 187), Se intakes of 200 or 100 μg/day led to 130 μg/L after only 6 or 3 months, respectively. Nevertheless, an additional Se intake of 200 μg/day through supplements is close to the recently established upper tolerable limit for Se long-term intake by the EFSA, 255 μg/day (51). In addition, as already mentioned, in US free-living men from California, with “normal” Se intake at the start, a diet with high Se intake (~200 μg/day for ~100 days) decreased concentration and total number of sperm, the proportion of motile sperm, and increased the proportion of tailless sperm (185). However, in that study, Se levels increased from 120 to 250 μg/L. Additionally, Se was given through meat and rice, and it is known that Se from dietary sources can be well absorbed and utilized. Nevertheless, in that study, only six participants were included in the high-Se arm, and it is possible that some other factors (including dietary) affected the results. Indeed, in another study in 22 USA free-living men by the same researchers, Se supplementation with even higher doses (300 μg per day as high-Se yeast for 48 weeks) did not affect serum androgen levels or semen quality (sperm count, motility, progressive motility, or morphology), despite increased serum and seminal plasma Se concentrations, from 140 to 230 μg/L, while sperm Se concentration even decreased (188). Nonetheless, in both those studies, the number of participants was quite low.

Overall, the currently available clinical studies/trials support the use of Se supplementation to improve male infertility, although high heterogeneity, insufficient numbers of participants, and lack of RCTs to control for possible confounding do not permit general recommendations to be made. Most studies also failed to take into account the most important clinical outcomes, which are pregnancy and live birth.

Therefore, more studies on the topic of the optimal dosing of Se, the optimal chemical form of the Se supplements, the optimal duration of Se supplementation, an optimal biomarker to follow, and its goal levels to achieve the optimal reproductive health effects in men need to be performed in the future.

Generally, if men are found to be deficient in Se, they could benefit from dietary or oral supplements. A reference range for Se in serum/plasma has been proposed (70–130 μg/L), since the plateau of maximal GPX activity (GPX3 in blood plasma; GPX1 in platelets and erythrocytes; and GPX4 in platelets) is achieved at Se intake of 40–60 μg/day and a plasma Se level of 70–100 μg/L. Nevertheless, the plateau for selenoprotein P in serum/plasma is achieved at higher levels, i.e., at Se intake of 60–70 μg/day and plasma Se levels of 90–130 μg/L (45, 51, 233). However, in light of the studies that indicate the increased risk for type 2 diabetes with higher Se intakes, probably the best range of Se in serum/plasma should be around 100–110 μg/L (57, 234, 235). In general, it has been suggested that Se serum levels < 60 μg/L can be considered “low Se status,” while Se levels from 60 to 80 μg/L can be considered a “gray zone” that could benefit from Se supplementation (90). However, we would like to point out that there is often a lack of consensus between different guidelines, and more research is needed, particularly on Se optimal levels in other body fluids/tissues (including seminal plasma or spermatozoa) or the status of other Se biomarkers in serum and other body fluids/tissues (45, 51, 57, 69). Furthermore, we would like to point out that it is still unclear whether seminal plasma, spermatozoa, or serum/plasma could provide more accurate information in terms of the role of Se in male infertility.

## 6 Integrative synthesis of the presented data and identification of research gaps

In summary, there is abundant literature evidence in animals and *in vitro* (less in humans) on the significant effect of Se on male infertility, with both Se deficiency and excess being connected with poorer reproductive health parameters, particularly in terms of seminogram characteristics. Selenium exerts its effects on reproductive health through both direct and indirect influences on the HPG axis and the reproductive tract, involving various selenoproteins at different stages.

However, the studies on the seminal plasma Se levels in men are limited, with particularly limited studies on the GPX levels/activity and selenoprotein P levels.

Overall, the studies indicate geographical variations in seminal Se levels (likely due to differences in soil and water Se content and dietary habits), typically lower seminal plasma Se levels in infertile men (but there are also instances of significantly higher levels, suggesting that Se excess could also be linked to infertility), positive associations of Se levels in seminal plasma and/or blood (serum/plasma) with seminogram parameters (particularly sperm motility), and support the use of Se supplementation to improve male idiopathic infertility.

Nevertheless, the number of studies on these topics is still limited, with varied quality, high heterogeneity, different methodologies (including variations in Se dosage, supplement formulations, intervention duration, and analytical techniques employed), and an insufficient number of participants. The possible confounding is also not well controlled.

Furthermore, there are just a few studies examining the effects of Se status or supplementation on outcomes of IVF and pregnancy, as well as the relationship between other functional Se-status biomarkers in seminal plasma (primarily GPX and selenoprotein P) and infertility.

More high-quality research is needed to determine the most suitable candidates for Se supplementation, optimal dosages, chemical formulations, duration of supplementation, and the best biomarkers of Se status related to reproductive health. Additionally, it is important to identify the most effective analytical techniques to assess these biomarkers, along with their optimal ranges. It is also essential to consider the risk of potential Se toxicity and adverse health effects associated with high-dose Se supplementation or elevated dietary intakes.

In general, Se supplementation could be considered in idiopathic infertile men with abnormal seminogram, particularly with low progressive motility, and with low Se intakes (< 60 μg/day) or serum levels (< 70 μg/L), but with doses in the range of 70–100 μg/day (maximum 200 μg/day), and probably in the form of Se-enriched yeast (which is the SeMet source) or L-SeMet, due to their lower toxicity. However, signs and symptoms of Se excess (selenosis) and serum Se levels should be monitored to adjust the doses and to avoid potential Se toxicity, particularly when higher doses of Se are prescribed (200 μg/day) with prolonged time (over 3 or 6 months). Unfortunately, at present, the routine Se measurements in blood/seminal plasma are unavailable in the majority of health services.

Additionally, as Se is also employed in female reproductive health, the studies that assess the outcomes of IVF and pregnancy should consider the role of Se in female fertility, taking into account Se intake, Se status (such as Se levels in serum/plasma or follicular fluid), or the effects of Se supplementation in female partners. However, studies on the role of Se in female fertility are rather scarce at present, and this area needs to be further explored.

## 7 Conclusion

In conclusion, the findings from studies involving experimental and domestic animals, cell cultures, and humans underscore the significant role of Se and selenoproteins in male fertility. These studies point to various mechanisms at play within different levels of the male reproductive system, emphasizing not only the contribution of Se and selenoproteins to the redox system, but also some more specific functions, including structural ones. Selenoproteins appear to play a crucial part not just in antioxidant defense but also in maintaining the integrity and functionality of spermatozoa. This suggests that these proteins are vital not only for protecting spermatozoa from oxidative stress but also for ensuring their proper development and performance, ultimately influencing male fertility.

Indeed, most research in humans demonstrates a positive relationship between Se levels in seminal plasma and blood with fertility and specific seminogram quality parameters, particularly sperm motility. Collectively, these findings, alongside limited clinical trials, support the potential benefits of Se supplementation for enhancing male infertility.

However, the studies in humans are still scarce and of varied quality, often with inadequate sample size, varied analytical techniques employed, and not well-controlled for possible confounding. Particularly scarce are studies on the correlation of other functional Se-status biomarkers (including various selenoproteins) in seminal plasma with infertility. Furthermore, the impact of Se excess must be considered due to potential toxicity and adverse effects on fertility, since U-shaped associations with infertility were shown.

Therefore, further well-designed studies are essential to confirm the role of Se in male infertility in humans and to identify optimal Se-status biomarkers related to male fertility, establish their ideal ranges, refine analytical techniques for assessment, and determine the appropriate dosages, formulations, and treatment durations for Se supplementation.
